# Interpretable Artificial Intelligence Decodes the Chemical Structural Essence of Twisted Intramolecular Charge Transfer and Planar Intramolecular Charge Transfer Fluorophores

**DOI:** 10.34133/research.1021

**Published:** 2025-12-09

**Authors:** Shuai Huang, Wenzhi Huang, Yanpeng Fang, Yingli Zhu, Jiaguo Huang, Fei Chen, Jie Dong, Wenbin Zeng

**Affiliations:** ^1^ Xiangya School of Pharmaceutical Sciences, Central South University, Changsha 410083, P. R. China.; ^2^ State Key Laboratory of Anti-Infective Drug Discovery and Development, School of Pharmaceutical Sciences, Sun Yat-sen University, Guangzhou 510000, P. R. China.

## Abstract

D–π–A-type fluorescent materials are crucial tools in life sciences and medicine, with their development hinging on a precise understanding of fluorophore mechanisms, particularly twisted intramolecular charge transfer (TICT) and planar intramolecular charge transfer (PICT) processes. These fluorophores exhibit unique charge transfer properties, making them highly valuable in organic optoelectronics, fluorescent probes, and sensors. However, despite their growing applications, the structural essence of TICT and PICT fluorophores remains poorly understood. This often results in molecules with similar structures displaying charge transfer modes that contradict design expectations, substantially hindering the application of TICT and PICT fluorescent probes. In this study, we meticulously designed various computational strategies based on interpretable machine learning to thoroughly deconstruct the chemical structural essence of TICT and PICT fluorophores. Utilizing the first real-world TICT and PICT dataset, we constructed predictive models that balance both interpretability and accuracy (area under the receiver operating characteristic curve = 0.846) using a range of algorithms, including deep learning. We established artificial intelligence (AI)-guided rules comprising 5 structural factors—electron-donating group strength, electron-withdrawing group strength, alkyl cyclization, steric hindrance, and solvent–solute interactions—that influence TICT and PICT. These rules provide obvious guidance for probe design based on molecular rigidity and charge transfer driving forces. Compared to community-suggested rules, the AI-guided rules achieved an over 20% improvement in accuracy in a controlled evaluation. By applying these rules, we successfully synthesized and validated several representative fluorophores that are challenging to distinguish using chemical intuition alone. Both quantitative calculations and experimental results confirmed the accuracy of the model and the practicality of the AI-guided rules. This novel approach is expected to establish a novel paradigm for exploring ideal TICT and PICT molecules, offering a robust framework for future research and application in fluorescent materials.

## Introduction

Owing to their unique optical characteristics and versatile functionalities, D–π–A-type fluorescent materials have attracted considerable attention in recent years. Fluorophores that emit from an excited-state intramolecular charge transfer (ICT) are prevalent [1–3], especially twisted intramolecular charge transfer (TICT) and planar intramolecular charge transfer (PICT) fluorophores, which exhibit excellent performance in applications such as organic optoelectronic materials, fluorescent probes, and sensors due to their unique charge transfer properties [4–6]. In this work, TICT and PICT fluorophores are defined to represent ICT systems without and with rotationally induced charge transfer, respectively. On excitation, the charge transfer in D–π–A-type compounds often proceeds with drastic conformational change; the bridge or covalent bond may twist or flatten, leading to TICT or PICT, respectively [7,8]. In the TICT process, the fluorophores undergo a conformational change from a quasi-planar emissive state to an almost perpendicular conformation through intramolecular bond rotation [9–11]. The fluorescence of the molecule gradually quenches with increasing degree of charge transfer during the locally excited to TICT transformation, which involves intermediate partially charge-transferred states retaining weak emission before reaching the non-emissive TICT state [12–14]. Therefore, TICT molecules are highly sensitive to their surrounding environment, including polarity, viscosity, and temperature [15–17]. To this end, tuning the TICT state in fluorophores has been utilized in many high-performance materials, such as polarity probes, viscosity sensors, and luminescent materials with aggregation-induced emission (AIE) [18–21]. In contrast, PICT fluorophores generate efficient and rapid ICT without intramolecular bond rotation, resulting in bright and stable fluorescence [22,23]. Thus, we anticipate the development of PICT dyes with sufficient brightness and photostability for applications in radiometric fluorescent probes, organic light-emitting diodes, etc. [24–26]. However, due to PICT fluorophores generating ICT without bond rotation, PICT dyes lose some of their potential as sensors [27]. Although both TICT and PICT belong to ICT, to distinguish whether a D–π–A-type fluorophore is TICT or PICT, we need to evaluate the frontier orbital interactions between the donor and the acceptor to effectively predict whether fluorophores planarize or twist in the excited state [28]. In other words, simply relying on the molecular structure or chemical intuition does not allow us to accurately distinguish whether the charge transfer in a specific molecule exhibits TICT or PICT, leading to these molecules with similar structures exhibiting charge transfer modes completely contrary to design [29], enormously increasing the trial-and-error costs in the development of fluorescent dyes and probes. Fluorescent dyes and probes are currently experiencing a rapid evolution from empirical trial-and-error strategies to rational molecular engineering [30–32]. In this evolution, the ability to precisely and quickly design D–π–A-type molecules as TICT or PICT with minimal cost is crucial for improving molecular design and developing high-performance TICT or PICT materials. Unfortunately, we currently lack powerful tools to assist chemists in the rapid and accurate design of TICT or PICT fluorophores.

Currently, quantitative calculation may serve as the only tool to visualize the TICT and PICT state [33]. Wang et al. [34] used time-dependent density functional theory to model 14 types of TICT organic fluorophores, establishing a reliable computational framework for modeling TICT formation. By using this approach, they designed a boron dipyrromethene-based AIE luminogen that exhibited nearly unhindered TICT rotation, leading to fluorescence quenching. In contrast, as TICT formation is inhibited in the molecular aggregate, this compound displayed bright emissions with 27-fold fluorescence. Zhong [35] also analyzed several classic TICT and PICT molecules through quantitative calculation from molecular structures, qualitative orbital interaction diagrams, and quantitative energy decomposition analyses, revealing the driving forces for TICT or PICT. Three driving forces were identified: the energy gap, hole–electron interactions, and excited-state relaxation. These studies have revealed the reasons behind TICT and PICT to some extent from the perspective of frontier molecular orbital theory. Indeed, over the past 2 decades, quantitative calculation has achieved undeniable success in the design, development, and improvement of fluorophores [36,37]. However, due to the high complexity of quantitative calculation, quantitative molecular design requires considering multiple factors, such as the diversity of fluorophore structures, embedded environments, and the choice of functionals. Misuse of quantitative calculation may lead to drastically different results, and due to their heavy computational demands, quantitative calculations are now primarily employed to offer supplementary insights into mechanisms.

The recent explosive growth of biomedical data with the rapid development of computational software and hardware and machine learning, including deep learning, has substantially enhanced efficiency and innovation in drug research and development [38,39]. Leveraging the powerful classification capabilities of artificial intelligence (AI), Qiu et al. [40] successfully developed a high-performance method based on a quantum-mechanics-aided machine learning algorithm to predict the AIE effect in triphenylamine fluorophores. Dong et al. [41] and Zhu et al. [42] employed AI algorithms to establish a novel multilevel framework for predicting organelle-targeted fluorescent probes. Through machine learning, they not only revealed the mechanisms by which fluorescent probes target organelles but also provided researchers with a new method to evaluate and design organelle-targeted fluorescent probes beyond chemical intuition. Additionally, Sumita et al. developed fluorescent molecules using a de novo molecule generator coupled with quantum chemical computation, successfully generating 3,643 candidate fluorescent molecules in 5 d. Their experimental validation showed that the generator’s accuracy in designing fluorescent molecules reached as high as 75% [43]. With this in mind, leveraging AI models may potentially overcome the current challenges in developing TICT and PICT, thereby reducing costs and improving efficiency in the development of fluorescent probes and dyes.

**Fig. 1.**
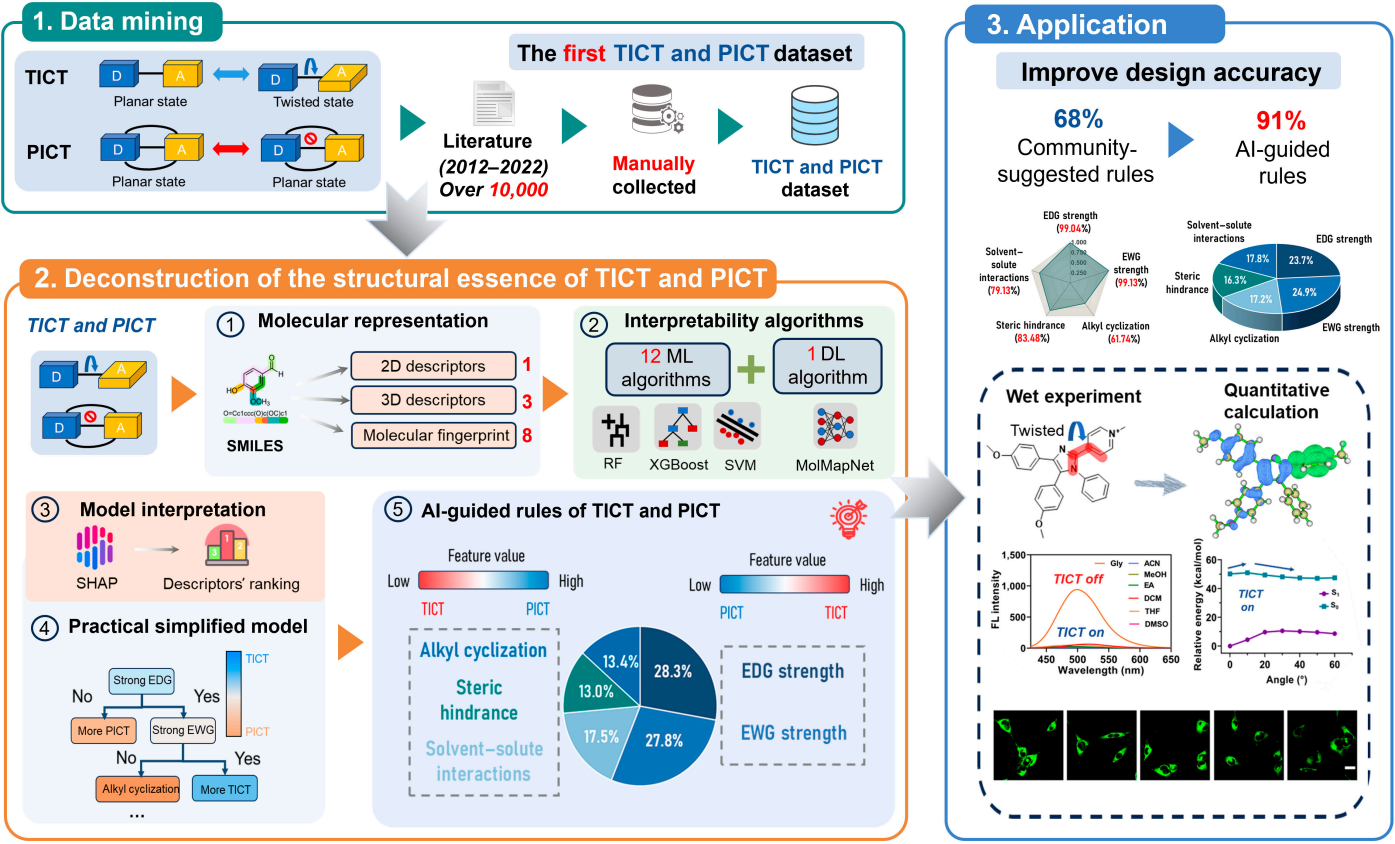
Workflow for interpretable machine learning deconstruction of the structural essence of twisted intramolecular charge transfer (TICT) and planar intramolecular charge transfer (PICT) fluorophores. SMILES, Simplified Molecular Input Line Entry System; 2D, 2-dimensional; 3D, 3-dimensional; ML, machine learning; DL, deep learning; RF, random forest; SVM, support vector machine; SHAP, Shapley additive explanations; EDG, electron-donating group; EWG, electron-withdrawing group; AI, artificial intelligence; FL, fluorescence; ACN, acetonitrile; MeOH, methanol; EA, ethyl acetate; DCM, dichloromethane; THF, tetrahydrofuran; DMSO, dimethyl sulfoxide; Gly, glycerol.

Here, we propose a novel strategy that integrates advanced interpretable machine learning with quantitative calculations to deconstruct the structural essence of TICT and PICT fluorophores, offering new insights into the precise design of fluorescent probes (Fig. 1). Unlike traditional quantitative computational methods, leveraging AI to deeply analyze the chemical nature of TICT and PICT requires addressing several key prerequisites: achieving a balance between accuracy and computational efficiency while establishing a rigorous mathematical relationship between molecular structural information and TICT and PICT properties. To this end, we first compiled chemical knowledge recognizable by AI from a vast array of real-world structures, constructing the first comprehensive TICT and PICT dataset spanning a decade (2012 to 2022). In the modeling process, we employed 12 traditional machine learning algorithms and 1 deep learning algorithm, ranging from simple to complex, to strike a balance between accuracy and interpretability. By analyzing various model contribution methods, we uncovered 5 design rules rooted in molecular rigidity and charge transfer driving forces that govern TICT and PICT behaviors. While these rules provide a groundbreaking perspective for designing TICT and PICT molecules, the complexity of the models may pose challenges for nonexperts to understand and apply. To address this, we developed a more intuitive and practical tool to visualize the stepwise differentiation of TICT and PICT molecules, facilitating real-world applications. Compared to those designed using community-suggested rules for probe design, TICT and PICT fluorophores designed using our AI-guided rules demonstrated an obvious improvement in accuracy. The applicability of our findings was further validated through experimental methods and quantitative calculations. By combining these innovative strategies, we have established a novel approach that enables researchers to design and control TICT and PICT states with greater precision and rationality, thereby advancing the field and opening new avenues for exploration.

## Results

### Construction of the TICT and PICT dataset

Currently, accurately identifying TICT and PICT probe molecules beyond experimental determinations remains challenging, making the availability of data relatively scarce. To ensure that our model is powerful enough, we manually collected data from over 10,000 publications spanning the last 10 years (2012 to 2022). We used the potential energy surface (PES) scan and corresponding conclusions from theoretical calculations reported in the publications as criteria to determine whether a molecule exhibits TICT or PICT behavior and initially gathered over 3,000 molecules. After the initial screening process, we ultimately identified 574 TICT molecules and 414 PICT molecules; their principal chemical scaffolds are listed in Fig. Supplementary materials. These molecules were firstly manually checked and then converted into InChIKey by using ChemDes and PyBioMed [44]. Molecules with the same InChIKey and label were deleted, while those with the same InChIKey but different labels were separated and retained. Ultimately, 511 molecules with TICT properties and 369 molecules with PICT properties were selected to build the dataset.

All molecules were calculated into 12 types of molecular representations: 1 type of 2-dimensional (2D) descriptor, 3 types of semiempirical 3-dimensional (3D) descriptors (Austin model 1 [AM1], parametric method 3 [PM3], and modified neglect of diatomic overlap [MNDO]), and 8 types of molecular fingerprints. The numbers of molecules for the 3 types of 3D descriptors were calculated and are listed in Table S1.

### Accurate prediction of TICT and PICT probes

**Fig. 2.**
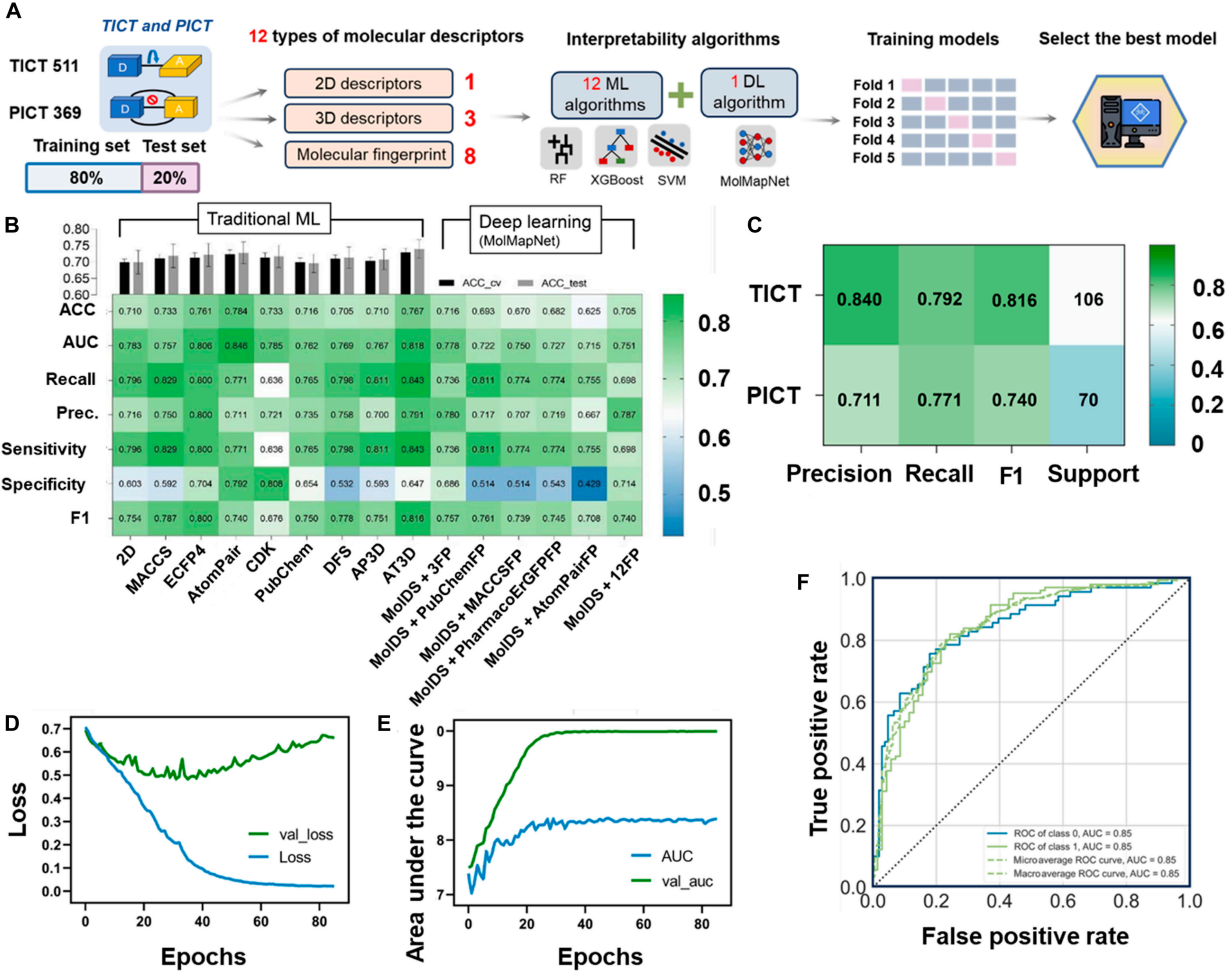
The performance of models by different molecular descriptors and algorithms. (A) The diagram illustrates the selection of molecular representations and algorithms in model construction to determine the optimal model. (B) The heatmap shows the performance of models constructed using different molecular representations on the test set, while the bar chart illustrates the differences in accuracy between the test set and cross-validation. In the MolMapNet results, MolDS represents the descriptor channel; 3FP refers to PubChemFP, MACCSFP, and PharmacoErGFP; and 12FP denotes all fingerprints in MolMapNet. (C) The classification report of the best model of XGBoost–AtomPair on the test set. “Support” represents the numbers of molecules in the test set. (D) The loss curve of the best model based on MolMapNet. (E) The area under the receiver operating characteristic (ROC) curve (AUC) of the best model based on MolMapNet. The model achieved an AUC of 0.834 during training process. (F) The ROC curve of the best model of XGBoost–AtomPair with an AUC of 0.846. ACC, accuracy; Prec., precision; val_loss, validation loss; val_auc, validation AUC.

We employed various AI algorithms combined with different molecular representations to establish a series of models to select the best model (Fig. 2A). First, traditional machine learning models were constructed by using 2D descriptors and 8 types of molecular fingerprints. For each type of molecular representation, the model was trained 50 times by different training set splits, and the best-performing model was selected from 12 algorithms each time (Table S2). As shown in Fig. 2B, XGBoost with AtomPair fingerprint achieved the best performance. The accuracy was 0.722 for the cross-validation (CV) and 0.784 for the test set, and the area under the receiver operating characteristic (ROC) curve (AUC) values for both sets were 0.85 (Fig. 2F). Notably, other models also achieved relatively good predictive performance, but they exhibited an imbalance in predicting the 2 types of probes in the test set, with a noticeable gap between sensitivity and specificity. In contrast, XGBoost with AtomPair fingerprint demonstrated a more balanced prediction of the 2 classes in the test set (Fig. 2C and Fig. S3), with sensitivity and specificity of 0.771 and 0.792, respectively. In combinations involving semiempirical 3D descriptors, the AM1, PM3, and MNDO methods all showed good performance, with the AM1 method performing the best, achieving an accuracy of 0.73 in the test set (Table S3).

To further improve predictive accuracy, we employed a popular deep learning framework, MolMapNet, which leverages convolutional neural networks (CNNs) to predict drug properties from a big-data and multifeature dimensional perspective. It is well suited for deep learning scenarios requiring model interpretability. After tuning the parameters for MolMapNet, we finalized a multichannel CNN model with ROC–AUC as the metric, tanh as the activation function, and uniform manifold approximation and projection as embedding algorithm. The final best model incorporated 13 types of continuous physicochemical and topological descriptors, as well as PubChemFP, MACCSFP, and PharmacoErGFP fingerprints. From the learning curve, it can be seen that this model achieved an AUC of 0.834 (Fig. 2D and E) and stopped learning after 35 epochs. In the test set, the model also demonstrated good predictive performance, with an overall accuracy of 0.716 and an AUC of 0.778 (Table S4). It is worth noting that MolMapNet’s performance was not as strong as that of the XGBoost–AtomPair model. This may be attributed to the deep learning models typically relying on large datasets to effectively train their parameters and enhance generalization capabilities. However, by integrating multiple descriptors, MolMapNet still provides complementary insights in terms of model interpretation. When all factors were considered, the XGBoost–AtomPair model consistently outperformed the others.

### Interpretable contribution of important structures to TICT and PICT

The contribution analysis of important structures pinpoints the key structures that most influence the predictions of TICT and PICT. While the feature importance may not be entirely consistent due to differences between the selected algorithms, the most important features always have relatively high scores. Therefore, we chose the best models based on 4 different perspectives of descriptors for further interpretative analysis.

**Fig. 3.**
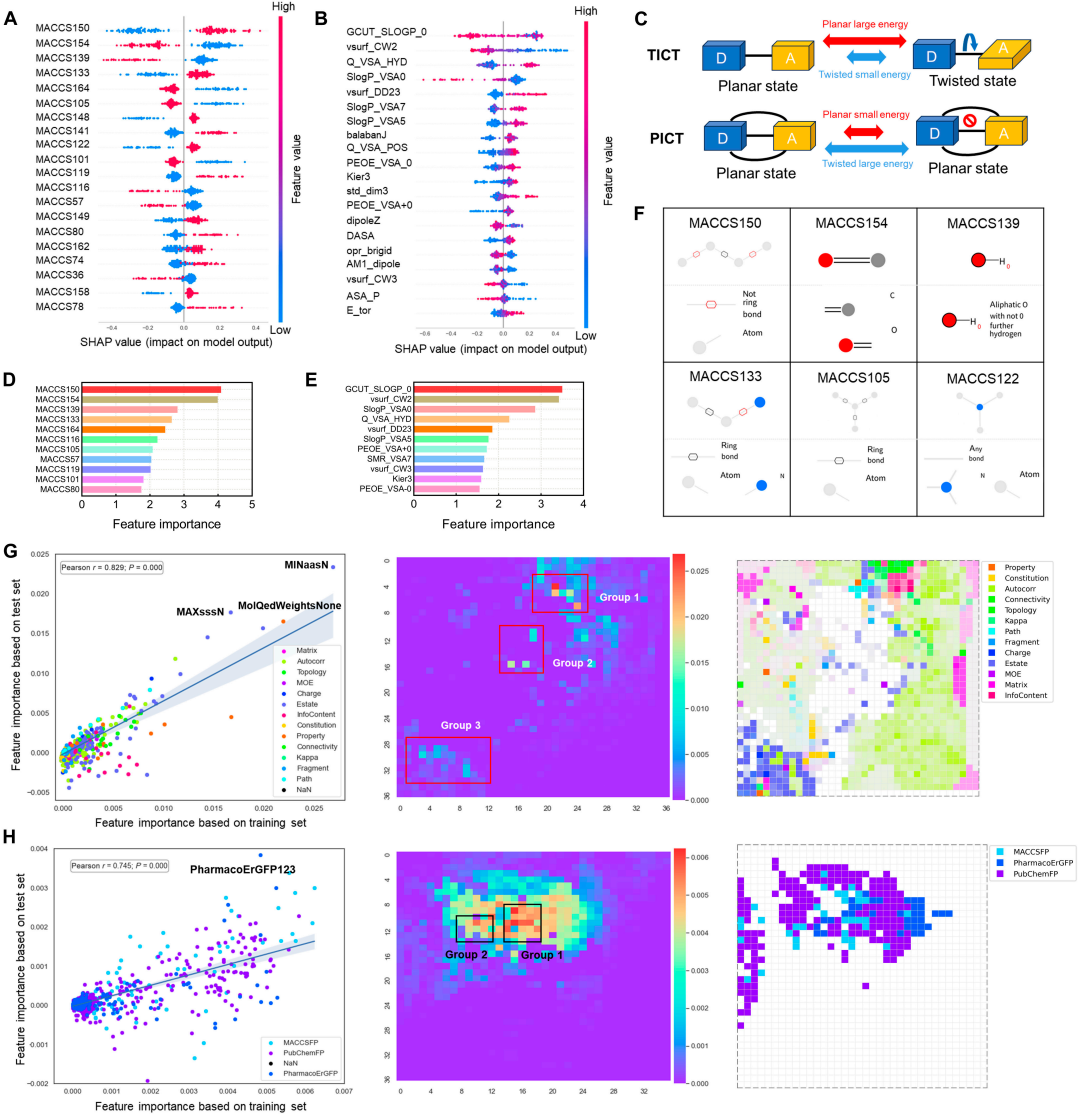
TICT and PICT model interpretation and structural analysis. (A) Top 20 feature importance based on SHAP values from the model with Molecular ACCess System (MACCS) fingerprint. (B) Top 20 feature importance based on SHAP values from the model with 2D + 3D descriptors. (C) Mechanism schematic of TICT and PICT. (D) Feature importance ranking by MACCS fingerprints. (E) Feature importance ranking by 2D + 3D descriptors. (F) Visualization of several important MACCS molecular fingerprints. (G and H) Model interpretation for the molecular fingerprint channel and descriptor channel of MolMapNet. The scatter plot on the left compares feature importance between the training set (*x* axis) and the test set (*y* axis); the heatmap on the right visualizes feature importance distribution in the feature space, with color intensity representing the importance score. Highlighted clusters indicate regions where features have similar impacts on the model’s performance.

Firstly, from the perspective of 2D descriptors, CatBoost–2D was the best combination that suits for interpretation. We selected 50 preprocessed 2D descriptors obtained through recursive feature elimination (RFE) and calculated their contributions to the model by using Shapley additive explanations (SHAP) (Fig. S5). Among them, GCUT_SLOGP_0 was identified as the most important descriptor, which is related to adjacency and distance matrices and partial equalization of orbital electronegativities (PEOE) partial charges. Additionally, SMR_VSA7, Kier3, PEOE_VSA-0, balabanJ, and h_pstates were also crucial descriptors for the model. These descriptors were related to molar refractivity, subdivided surface area, molecular shape, etc. Considering the differences in mechanisms and real-world application scenarios of TICT and PICT molecules, their planar and rotational energies are different (Fig. 3C); 3D descriptors that include spatial structural information may offer insights from a 3D perspective.

Secondly, from the perspective of 3D descriptors, it was also observed that after incorporating 3D descriptors, the previously identified important 2D descriptors still retained high scores. At the same time, we also found several important 3D descriptors. Specifically, vsurf_CW2, vsurf_CW3, and vsurf_DD23 were identified as key features calculated by the AM1 method. These descriptors are associated with molecular surface area, volume, and shape. Another set of important descriptors, dipoleZ, DASA, and AM1_dipole, was highly dependent on molecular conformation and represented charge distribution within the molecule. Additionally, descriptors calculated by the PM3 and MNDO methods, such as E_vdw (van der Waals energy) and E_tor (torsional energy), captured the effects of molecular strain and flexibility on electronic transitions (Fig. 3B and E and Figs. S6 and S7).

Thirdly, observing key molecular substructures, we interpreted the CatBoost–Molecular ACCess System (MACCS) combination. Although the XGBoost–AtomPair model exhibited the best predictive performance, the AtomPair fingerprint is less intuitive for structural interpretation compared to MACCS. Therefore, we selected the CatBoost–MACCS combination, the best-performing model among those based on the MACCS fingerprint. Notably, 45 out of the top 50 important substructures identified by both feature importance ranking and SHAP appeared repeatedly. MACCS150, MACCS154, MACCS139, MACCS133, and MACCS164 were identified as the top 5 most important features (Fig. 3A and D). From the SHAP methods, it can be observed that the presence of substructures MACCS150, MACCS133, MACCS148, and MACCS141 positively influenced the TICT properties of the molecules, while the presence of MACCS154, MACCS139, MACCS164, and MACCS105 negatively impacted TICT properties. Figure 3F visualizes some of these important substructures, while a comprehensive visualization of all key MACCS fingerprints is shown in Fig. S9.

Finally, although MolMapNet’s predictive performance was slightly lower than that of the best model, its deep learning framework allows the simultaneous integration of multiple descriptors and fingerprint types (Fig. 3G and H). Among the 1,369 descriptor features, most had relatively low importance scores, with 12 of the top 20 as Estate (Electro-topological State Atom Type) descriptors. The most important among these were MINaasN, MAXsssN, MAXaasN, and MINsssN, all related to nitrogen atom charges, while autocorrelation descriptors also greatly influenced the model. For the 1,303 fingerprint features, contributions were concentrated in the top 200 features, with 128 PubChemFP features accounting for 47.30% of the total importance score. MACCS and PharmacoErGFP followed, contributing 24.74% and 9.51%, respectively. Among these, PharmacoErGFP123, PharmacoErGFP124, PharmacoErGFP404, and PharmacoErGFP125—primarily linked to aromaticity—stood out as the most important, while MACCS158, MACCS148, MACCS150, MACCS133, MACCS122, MACCS149, MACCS156, and MACCS165 also played key roles (Fig. S8). The molecular representation information is shown in Tables S5 to S13.

### Practical simplified model based on interpretable descriptors

**Fig. 4.**
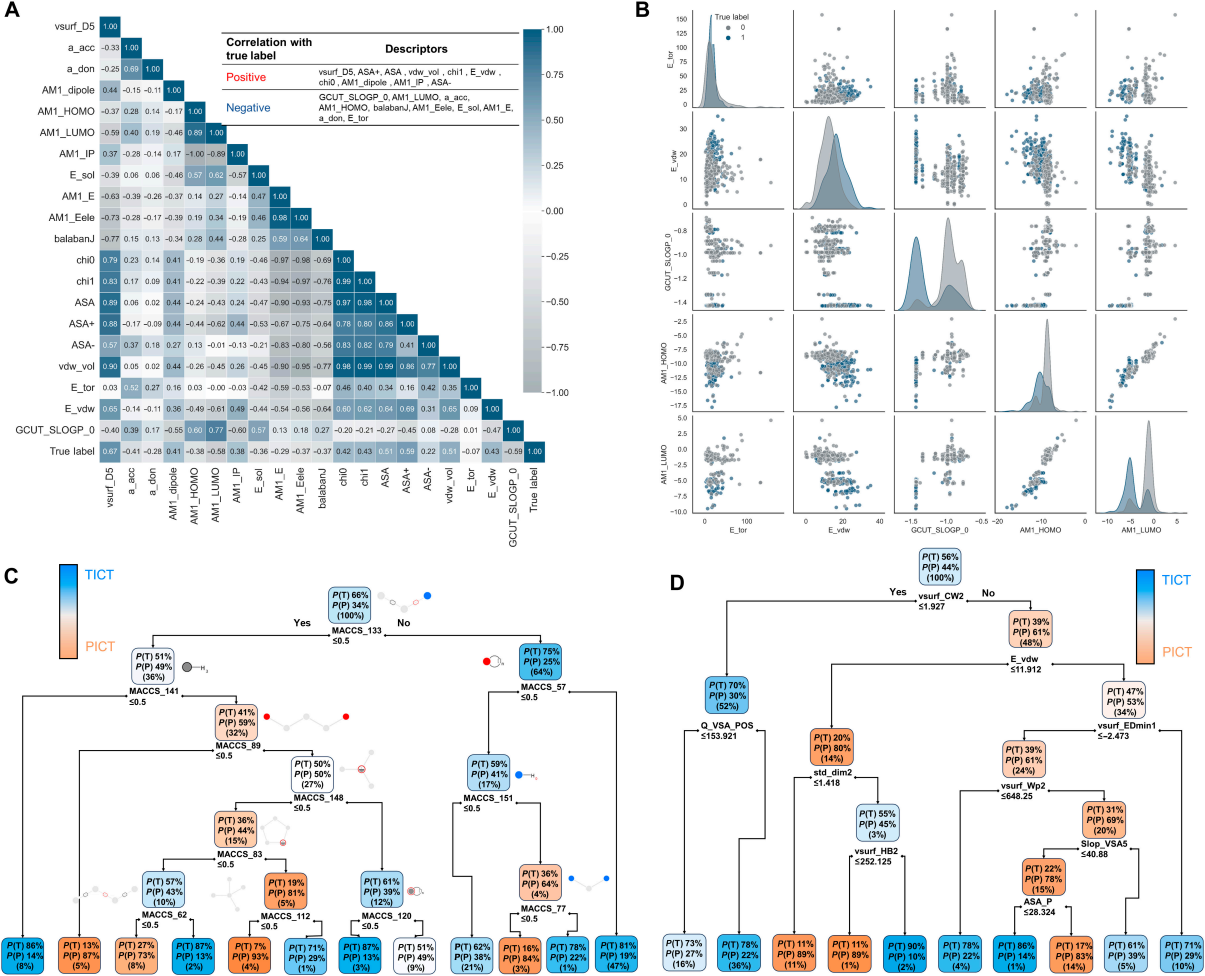
Analysis based on important descriptors and visualization of simplified decision tree models. (A) The top 20 descriptors most correlated with the true labels and their interfeature correlations. (B) Sample distribution of 5 important descriptors. (C) Visualization of the decision tree model constructed by MACCS fingerprints. (D) Visualization of the decision tree model constructed by 2D + 3D descriptors. *P*(T) and *P*(P), probabilities of a molecule being predicted as TICT or PICT, respectively.

To facilitate the practicality of our findings, a streamlined and practical model based on the interpretable descriptors was established. Figure 4A displays the top 20 features most correlated with the true labels. We found that whether the descriptors were positively or negatively correlated with the true labels, the best correlation reached 0.67. This relatively high correlation demonstrates the consistency of the interpretation of the model. Furthermore, the distribution of samples across 5 key descriptors shows that while there is some correlation between individual descriptors and TICT or PICT, relying on just one or a few descriptors is insufficient for accurate prediction (Fig. 4B). Recognizing the limitations of single descriptors in accurately distinguishing TICT from PICT, we constructed a series of simpler decision tree (DT) models based on MACCS fingerprints and 2D + 3D descriptors to facilitate researchers’ more direct understanding of how the model gradually distinguishes between TICT and PICT (Fig. 4C and D). In these models, *P*(T) and *P*(P) represent the probabilities of a molecule being predicted as TICT or PICT, respectively. The percentages in parentheses indicate the proportion of samples at each node relative to the total dataset. The DT model by MACCS fingerprints achieved an accuracy of 0.724 and 0.710 for the CV and test, respectively. The DT with 2D + 3D descriptors got lower accuracies than the DT with MACCS fingerprint, with CV and test accuracies of 0.646 and 0.647, respectively. Both of the simplified DT models provide a balance between interpretability and performance, making them valuable tools for quick applications.

### Deconstruction of the structural essence of TICT and PICT

To deeply explore the mechanisms of TICT and PICT, we deconstructed the structural essence of TICT and PICT from the perspective of key descriptors. We found that many descriptors are related to alkyl cyclization, steric hindrance, and alterations in the interactions between the fluorophore and the solvent. When these factors are enhanced in a fluorophore, they tend to favor PICT, and the reverse trend favors TICT. These findings align with some of the previously reported rules derived from chemical intuition [9]. Interestingly, we also observed that some descriptors related to charge and energy are also important for TICT and PICT, which are highly correlated with electron-donating group (EDG) and electron-withdrawing group (EWG) strengths. When the EDG and EWG strengths increase, the compound tends to exhibit TICT, and the reverse favors PICT. In other words, TICT will not occur even under favorable external conditions (e.g., steric hindrance and solvent effects) if the EDG/EWG strengths are insufficient. Finally, 5 key rules derived from scientific computational analysis were summarized: EDG strength, EWG strength, alkyl cyclization, steric hindrance, and solvent–solute interactions.

**Fig. 5.**
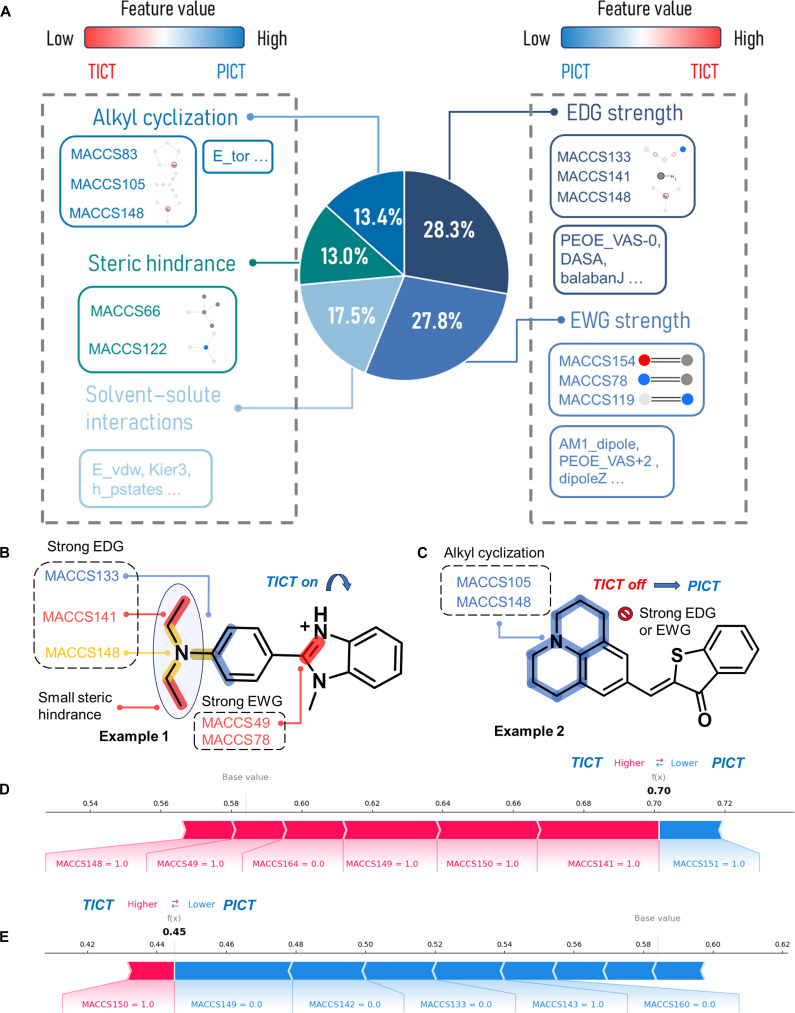
The schematic diagram of deconstructing the structural essence of TICT and PICT. (A) Proportion of model contribution from the 5 key factors. Notably, a very small number of features that recur across different groups (e.g., MACCS148) may assume different roles when the surrounding atomic environments vary. (B) Deconstructing the structural essence of TICT in example 1. (C) Deconstructing the structural essence of PICT in example 2. (D) Conducted SHAP force diagram analysis on the example of a TICT molecule by the MACCS–RF model. (E) Conducted SHAP force diagram analysis on the example of a PICT molecule by the MACCS–RF model.

Next, we systematically analyze how these descriptors influence the TICT and PICT process. The 5 categories of AI-guided rules were summarized based on the molecular descriptors selected from the best-performing models in this study. Specifically, descriptors calculated using 2D features, MACCS fingerprints, and 3D descriptors obtained from the AM1 and MNDO methods were derived from the CatBoost model, while descriptors calculated using the PM3 method were derived from the extra trees model. After combining and removing duplicates, a total of 36 descriptors were included in the analysis: 9 descriptors related to EDG strength, 9 related to EWG strength, 5 related to steric hindrance, 5 related to solvent–solute interactions, and 8 related to alkyl cyclization. Among these fragments, MACCS133, MACCS141, and MACCS148 can combine into a dimethylamino group, and with MACCS150, which is widely used as an EDG in conventional TICT fluorophores. Both MACCS105 and MACCS148 are also critical structural fragments. They represent molecular cyclization and bulky steric groups, respectively, while increasing the steric of the EDG also reduces interactions between the fluorophore and the solvent. Additionally, many EWGs, such as carbonyl (MACCS154) and cyano (MACCS78), could reduce the strength of the EDG. Figure 5A shows the proportion of model contribution calculated by SHAP from descriptors corresponding to the 5 rules. It can be observed that EDG strength and EWG strength account for a relatively large share, contributing 28.3% and 27.8%, respectively. As the EDG strength and EWG strength increase, the electron transfer capability in the compound also intensifies, making it more prone to the TICT process. Conversely, when these strengths decrease, the compound tends to favor the PICT process. In contrast, the contributions of alkyl cyclization, steric hindrance, and solvent–solute interactions are relatively smaller, accounting for 13.4%, 13.0%, and 17.5%, respectively. Alkyl cyclization could influence intramolecular electron transfer pathways by altering molecular flexibility and conformation. Steric hindrance introduces spatial constraints, limiting intermolecular interactions, which can stabilize or inhibit specific electron transfer processes. On the other hand, solvent–solute interactions indicate that factors such as solvent polarity and solute solubility can influence the electron state or conformation of the molecule, altering its preference for TICT or PICT. These 5 key factors can serve as AI-guided rules in future TICT and PICT molecular design.

To further provide a more straightforward explanation of the AI-guided rules, we selected one TICT molecule and one PICT molecule from the training set for analysis, both of which were representative examples of TICT and PICT molecules (Fig. 5B and C). Here, we selected the MACCS–random forest model, which combines strong interpretability and high predictive accuracy, to perform a SHAP force plot analysis on these 2 molecules. In the force plot, a SHAP value closer to 1 indicates that the molecule is more likely to be a TICT molecule, while a SHAP value closer to 0 indicates a higher trend to a PICT molecule. In the force diagrams, each segment reflects the contribution of specific structural fragments to the prediction outcomes. Here, MACCS = 1 indicates the presence of a contributing fragment, while MACCS = 0 indicates its absence. Highly influential fragments are visually highlighted and labeled in the molecular structures. As shown in Fig. 5B and D, in example 1, there is a strong EDG composed of MACCS133, MACCS141, and MACCS148, as well as a strong EWG composed of MACCS49 and MACCS78, which are conducive to electron flow and thus more favorable to TICT. Furthermore, the EDG in example 1 has minimal steric hindrance, leading to its classification as a TICT (SHAP value = 0.70) molecule. Similarly, in Fig. 5C and E, the EDG in example 2 is cyclized by an alkyl group (MACCS105 and MACCS148), and it lacks strong EDGs or EWGs, resulting in example 2 being classified as a PICT (SHAP value = 0.45) molecule. This aligns with the true reality of these 2 molecules. It should be noted that for an individual sample, the key feature contributions determining its classification (e.g., Fig. 5D and E) may not fully align with the global rules, which is not unexpected.

### Validation of AI-guided rules in TICT and PICT probe design

To validate the practicality of the AI-guided rules, we designed 2 TICT and PICT molecular libraries: one based on community-suggested rules and the other utilizing AI-guided rules. We conducted a 3-level systematic evaluation to assess the differences between the 2 design strategies. The first level involved comparing the basic physicochemical properties of the 2 libraries to ensure their comparability. The second level focused on design accuracy. The third level validated the results through a wet experiment and quantitative calculation.

**Fig. 6.**
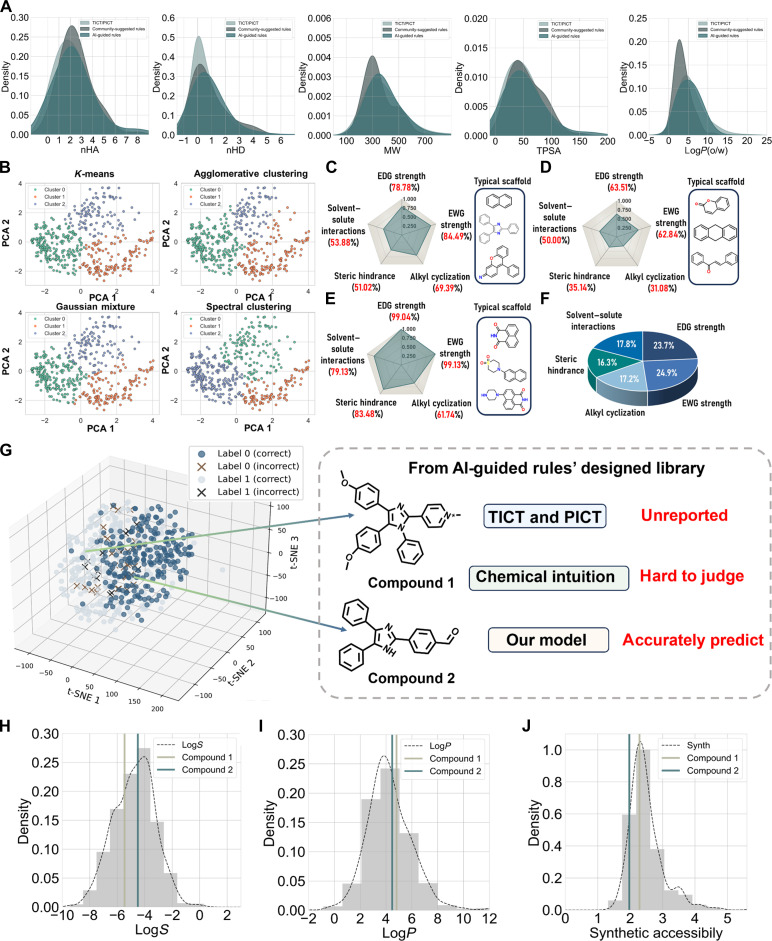
The validation results of AI-guided rules in the design of TICT and PICT probes. (A) The basic physicochemical properties of the AI-guided molecular library, the community-suggested molecular library, and the real TICT and PICT dataset. nHA and nHD indicate the counts of hydrogen-bond acceptors and donors, respectively. TPSA is a descriptor reflecting molecular polarity, Log*P* measures the oil–water partition coefficient, and MW represents the molecular weight. (B) Four clustering results are based on AI-guided molecular library, *K*-means, agglomerative clustering, Gaussian mixture, and spectral clustering. (C to E) The 3 categories are based on *K*-means clustering results, along with their proportions of AI-guided rules usage, each featuring several typical scaffolds. (F) Overall proportions of the rules utilized in the AI-guided molecular library. (G) Predicted distribution of AI-guided library after t-distributed stochastic neighbor embedding (t-SNE) dimensionality reduction (label 0: PICT; label 1: TICT). Compound 1 and compound 2 are 2 representative fluorophores whose TICT and PICT properties have not been reported. Their behavior is difficult to judge based on chemical intuition alone, but our model accurately predicts their properties. (H to J) Distribution of Log*S*, Log*P*, and synthesizability in the set of 881 TICT and PICT molecules. PCA, principal component analysis.

The molecular library based on community-suggested rules was designed by existing chemical intuition and structure–property relationships, representing the most common rules for designing TICT and PICT probes [9], which contains a total of 83 molecules. While the AI-guided rules library consists of 509 molecules. In the first level, we compared the basic physicochemical properties of the AI-guided molecular library, the community-suggested molecular library, and the real TICT and PICT dataset (Fig. 6A). The results show the 2 designed libraries exhibit highly consistent properties with the real dataset in terms of fundamental chemical space. Then, we further analyzed the distribution and differences of these molecules in the AI-guided molecular library and summarized their characteristics. We applied principal component analysis to reduce data dimensionality to minimize complexity and applied 4 different clustering methods, *K*-means, agglomerative clustering, Gaussian mixture, and spectral clustering, ultimately grouping the molecules into 3 categories, which maximized the separation between categories while maintaining intracluster compactness, avoiding excessive clustering that might lead to category overlap or noise (Fig. 6B). *K*-means achieved the best clustering performance. Consequently, we conducted a structural feature analysis of each category of molecules and showed the typical scaffolds of each category (Fig. 6C to E). Specifically, when summarizing all 3 categories of molecules, EDG and EWG strengths accounted for the largest proportions at 23.7% and 24.9%, respectively, while alkyl cyclization, steric hindrance, and solvent–solute interactions each remained around 17% (Fig. 6F). This similarity in proportion to the structural essence demonstrates that the design of our library strictly adheres to the AI-guided rules. In the second level, the molecules from these 2 libraries were fed into our best model to predict whether these compounds exhibit a TICT or a PICT state. The prediction results are shown in Fig. 6G and Fig. S10. The library based on community-suggested rules got an accuracy of 67.47%. It seems to be a reasonable accuracy in designing TICT or PICT molecules, but there is still potential for further improvement. In contrast, the AI-guided library achieved an accuracy of 90.96%, improving by more than 20% compared to the current state-of-the-art approach for precise probe design.

However, the prediction results reveal that molecules with highly similar structures may exhibit distinctly different outcomes. In the third level, we selected 2 fluorophores with unreported TICT or PICT properties, compound 1 and compound 2, whose mechanisms are hard to judge by chemical intuition but were accurately predicted by our model, for further validation (Fig. 6G). Thus, we utilized the ADMETlab platform [45] to evaluate the physicochemical properties and synthetic accessibility of these compounds. Among the various characteristics provided by ADMETlab 2.0 [46], Log*S* and Log*P* serve as key indicators of compound solubility in water and lipids, respectively. Additionally, synthetic accessibility provides a balanced measure of feasibility and cost. Figure 6H to J shows their distribution of Log*S*, Log*P*, and synthetic accessibility. Both of the 2 compounds have Log*S* and Log*P* values in the optimal range for drug-like molecules and have an acceptable synthetic difficulty. We aim to validate the effectiveness of AI-guided rules through quantitative calculation and wet experiments on these 2 molecules.

### Theoretical calculation

**Fig. 7.**
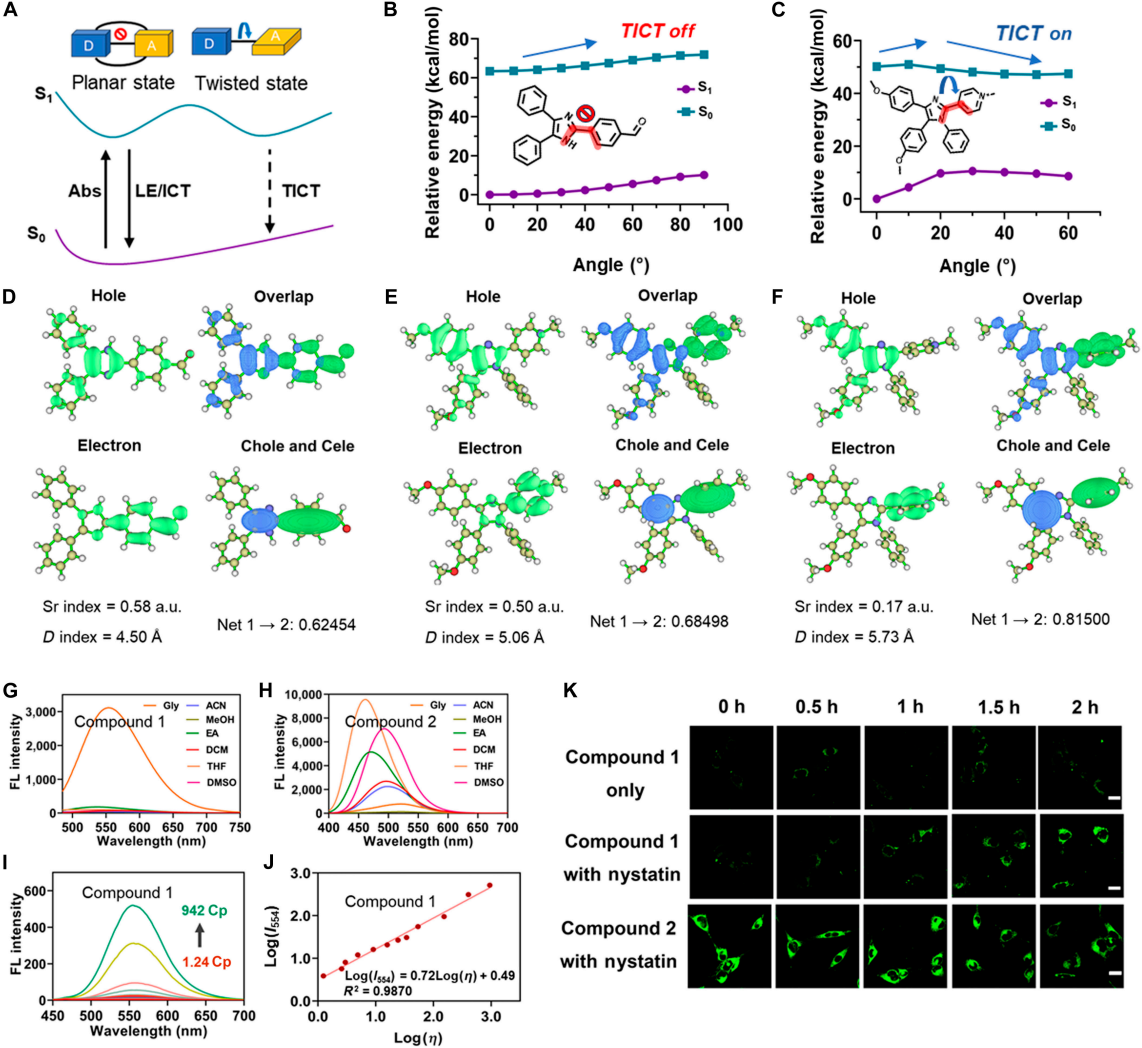
Validation results of compound 1 and compound 2. (A) Schematic diagram of the TICT mechanism. (B and C) The potential energy curves of TICT corresponding to compound 2 and compound 1, respectively. The ground-state structure of all compounds was optimized using the Gaussian 16 software at the PBE0/6-31G* level of theory, and their excited states were calculated by TD-PBE0/6-31G*. (D) Electron–hole analysis of compound 2 in the hybrid local and charge transfer (HLCT) state. Sr (spatial overlap) quantifies the degree of spatial overlap between the electron and hole densities; a smaller Sr indicates a stronger charge transfer character. *D* (electron–hole centroid distance) represents the distance between the centroids of the electron and hole densities, with a larger *D* corresponding to more pronounced charge separation. Net 1 → 2 (net charge transferred) denotes the net amount of charge transferred from the hole to the electron upon excitation. (E and F) Electron–hole analysis of compound 1 in the PICT and TICT states, respectively. The single-point energy was calculated at the TD-PBE0/Def2-TZVP level. (G and H) Emission spectra of compound 1 and compound 2 in different solvents. (I) Fluorescence intensity response of compound 1 at 414-nm excitation as a function of viscosity. (J) Relationship between fluorescence intensity and viscosity for compound 1. (K) Imaging of the viscosity response of compound 1 and compound 2 in cells. The scale bar is 20 μm. LE, locally excited; ICT, intramolecular charge transfer; Chole, hole contribution; Cele, electron contribution.

For TICT molecules, excited-state PES scans serve as a crucial qualitative method (Fig. 7A). After obtaining the most stable ground-state conformations of each compound, we conducted PES scans of their dihedral angles. For compound 1, we scanned the π–A dihedral angle, finding that when this angle approaches 90°, the S_1_ state reaches its lowest energy. This suggests that upon excitation, the molecule only needs to overcome a barrier of 0.79 kcal/mol to relax to a near-90° conformation and can quickly reach the TICT state (Fig. 7C). For compound 2, the PES scan revealed that as the dihedral angle increases, the barrier of the S_1_ state also increases (Fig. 7B). This implies that this molecule cannot reach the dark TICT state on excitation. Further analysis of the electron–hole properties after excitation showed that compound 2 exhibits a clear hybrid local and charge transfer (HLCT) state with an oscillator strength of 0.81 (Fig. S13), confirming a bright state. On the other hand, compound 1 exhibits HLCT characteristics in the PICT state with an oscillator strength of 0.44, also indicating a bright state. When reaching the TICT state, it displays a fully charge-separated configuration with an oscillator strength of 0 (Fig. S13), indicating a dark state. Notably, electron–hole analysis calculations indicate that molecules undergo more extensive ICT in the TICT state, with longer electron transfer distances and higher transfer quantities (Fig. 7D to F).

### Synthesis of compound 1 and compound 2

The detailed synthesis process and nuclear magnetic resonance and high-resolution mass spectrometry analysis can be found in the Supplementary Materials.

### Spectroscopic properties of compound 1 and compound 2

After obtaining compound 1 and compound 2, we first measured the ultraviolet spectra of compound 1 and compound 2 in different solvents (Table S14). As shown in Figs. S11 and S12, compound 1 exhibits a distinct absorption band with a peak of around 414 nm, whereas compound 2 shows an absorption peak of around 360 nm. Subsequently, we measured the emission spectrum of the 2 compounds in different solvents. As seen in Fig. 7G and H, compound 1 shows strong fluorescence only in glycerol (554 nm), while the fluorescence intensity of compound 2 varies with solvent polarity, indicating that compound 1 responds to viscosity rather than polarity. Next, we conducted a quantitative evaluation of the viscosity response of compound 1 in mixed solvents of methanol and glycerol at different ratios. In Fig. 7I, the fluorescence intensity at 554 nm increases as the proportion of glycerol rises, ultimately enhancing the fluorescence intensity 133-fold as the viscosity increases from 1.24 to 942 cP. More importantly, good linearity was displayed between Log(*I*_554_) and Log(*η*) based on a Förster–Hoffmann equation of Log(*I*_554_) = 0.7260Log(*η*) + 0.4931, the linear coefficient is 0.9870 (Fig. 7J). This confirms that compound 1 has viscosity-sensitive characteristics, where an increase in viscosity restricts intramolecular bond rotation, thus inhibiting TICT and enhancing fluorescence emission. The spectral properties of compound 2 suggest that fluorescence emission occurs without the need for intramolecular bond rotation, aligning with the PICT mechanism. Generally, the above results are consistent with the quantitative calculation, which established a reliable groundwork for the subsequent imaging investigations.

### Monitoring the variations in mitochondrial viscosity in living cells

Currently, many TICT-based fluorescent probes are used to detect intracellular viscosity changes [47–49]. Following the successful in vitro responses of the 2 compounds, additional cellular imaging experiments were performed to validate the proposed mechanisms of compound 1 and compound 2. We investigated their ability to detect viscosity changes in cells by Hepa1-6 cells for bioimaging. It has been reported that nystatin can induce structural changes and swelling in mitochondria, thereby increasing intracellular viscosity [50]. Therefore, nystatin can be used as a viscosity inducer to evaluate the visualization of mitochondrial viscosity in Hepa1-6 cells. After incubating Hepa1-6 cells with 10 μM compound 1 and compound 2 for 30 min, we added 10 μM nystatin and continued incubation. As shown in Fig. 7K and Fig. S15, the fluorescence intensity of compound 1 with nystatin gradually increased over time compared to that of compound 1 alone. This indicates that compound 1 is also responsive to viscosity in live cells. In contrast, compound 2 showed a high fluorescence intensity without nystatin incubation, and its fluorescence intensity did not change obviously with prolonged incubation with nystatin. These comprehensive experiments confirm that compound 1 exhibits TICT, while compound 2 exhibits PICT, successfully validating the accuracy of our AI model in predicting the TICT and PICT molecules.

## Discussion

AI-based prediction of TICT and PICT differs evidently from other conventional molecular properties’ predictions. TICT and PICT involve complex electronic interactions and dynamic behavior, unlike properties such as polarity or hydrophilicity, which can often be predicted using simpler descriptors. However, TICT and PICT prediction faces challenges in terms of molecular standards, method selection, and even data processing.

Since, as far as we know, TICT and PICT prediction models have not been reported yet, we were unable to make direct comparisons with similar models. During the modeling process, we conducted a comprehensive comparison of descriptor attempts, model balance, and the cost-effectiveness of the models. This is also why many of the attempted results were not ultimately selected. The AtomPair fingerprints with the XGBoost algorithm exhibited the best performance, with CV and test set accuracies of 0.722 and 0.784, respectively. Notably, although other combinations also have good performance, they showed an imbalance in predicting the 2 types of probes in the test set, with a noticeable gap in sensitivity and specificity. In contrast, the combination of AtomPair fingerprints with the XGBoost algorithm not only achieved the best predictive performance but also maintained a balanced prediction, with sensitivity and specificity of 0.771 and 0.792, respectively. Beyond the combination of algorithms and descriptors, the 3D information and the convolutional methods for extracting features also affected model performance. After incorporating 3D descriptors, the model’s performance did not show obvious improvement. Considering the computational cost, we believe that this model is not suitable for large-scale calculations in the future. Additionally, MolMapNet did not perform as well as the previous best model, but it still achieved a decent prediction accuracy of 0.716 and showed a good reclassification balance. It cannot be denied that as the data size increases, CNN models may achieve more robust performance.

The complex mechanisms of TICT and PICT still pose challenges for chemists, but benefiting from the model’s strong interpretability, we deconstruct these core substructures and physicochemical properties that influence the TICT and PICT processes, which can be summarized into 5 AI-guided rules: EDG strength, EWG strength, alkyl cyclization, steric hindrance, and solvent–solute interactions. These rules can not only be explained from an algorithmic perspective but also effectively be linked to chemical mechanisms. The TICT process typically requires dramatic conformational twisting of the molecule in the excited state. This twisting helps to minimize π–π orbital overlap between the donor and acceptor regions, resulting in prominent intramolecular charge separation. In contrast, the PICT process tends to maintain the planar configuration between the donor and acceptor to enhance π–π electronic conjugation and orbital overlap. Specifically, stronger EDGs enhance the electron-donating capability toward the acceptor region and, in combination with strong EWGs, effectively facilitate the redistribution of electronic density. Due to TICT’s reliance on rapid intramolecular charge separation, strong EDGs and EWGs provide sufficient driving force for charge transfer. Conversely, weaker EDGs and EWGs reduce the electronic density difference between the donor and the acceptor, lowering the driving force for twisting and preserving molecular planarity, which is favorable for forming a stable PICT state. Besides EDG and EWG strengths, alkyl cyclization, steric hindrance, and solvent–solute interactions also play important roles in regulating molecular twisting and planarity in TICT and PICT processes. Alkyl cyclization primarily affects conformational changes by modulating molecular rigidity. Cyclized molecules typically exhibit higher rigidity and planarity. This rigidity can restrict the rotation of molecular fragments, thereby suppressing TICT and promoting PICT. However, excessive alkyl cyclization might disrupt molecular planarity and disturb the conjugated system, reducing PICT efficiency. The mechanisms of steric hindrance and solvent–solute interactions are similar, as both influence the rotational freedom of molecular fragments to regulate TICT and PICT processes. Smaller steric hindrance and highly polar solvents lower rotational barriers, facilitating conformational twisting between donor and acceptor regions, thus favoring the TICT process. In contrast, larger steric hindrance and low-polarity solvents limit rotational freedom, maintaining molecular planarity and stabilizing π–π conjugation, which supports the formation of a stable PICT state. The key substructures may provide a more intuitive explanation of factors affecting TICT and PICT. From a thermodynamic perspective, the traditional chemical design of TICT and PICT molecules often overlooks the heavy dependency of ICT on the strength of EDGs and EWGs. For example, dialkylamino groups composed of MACCS133, MACCS141, and MACCS148 exhibit strong electron-donating capabilities and are among the most commonly used EDGs in TICT systems. The EWGs corresponding to fragments such as MACCS78, MACCS119, and MACCS154 possess strong electron-withdrawing abilities, thereby greatly facilitating electron flow and the formation and stabilization of the TICT state. From a kinetic perspective, MACCS105 and MACCS148 can regulate the alkyl cyclization of EDGs and increase steric hindrance to control TICT and PICT states. For example, when the EDG is a dimethylamino group, it possesses strong electron-donating ability and can rotate in the excited state to achieve TICT. However, as the alkyl chain lengthens or cyclizes, these changes directly inhibit intramolecular bond rotation, thereby suppressing the TICT process and instead favoring the formation of the PICT state. It is worth noting that it is challenging to directly observe the conformational and charge-related properties of molecules, which makes it difficult to quickly and accurately distinguish TICT from PICT based solely on chemical intuition. However, our models could rapidly and accurately predict TICT and PICT molecules, markedly improving the efficiency of designing TICT and PICT molecules. Recognizing that not all researchers may be familiar with complex models, we also developed a simplified version that balances both accuracy and interpretability to make it more accessible for researchers to apply in real-world design scenarios.

The effectiveness of AI-guided rules is crucial for their practical application. In validating the practical application of the AI-guided rules for TICT and PICT, the molecular library designed based on AI-guided rules achieved an accuracy 23.5% higher than that of the library based on community-suggested rules in a similar chemical space. It was indicated that even on the foundation of existing precise design strategies, our AI-guided rules can still greatly enhance the design efficiency of TICT and PICT probes. This result was fully validated through both quantitative calculations and wet experiments. According to our quantitative calculation results for the 2 representative fluorophores, the PES curve shows that compound 1 requires a much lower energy barrier to undergo TICT compared to compound 2. Moreover, the electron–hole analysis reveals that the oscillator strength for compound 1 in the TICT state is 0, while for compound 2 it is 0.81, and the charge transfer amount for compound 1 in the TICT state is greater than that in its PICT state. All of these findings support the conclusion that compound 1 is a TICT molecule, whereas compound 2 is a PICT molecule. This view was also validated experimentally: when external conditions restricted the rotation of compound 1, its fluorescence intensity increased, whereas compound 2 showed no such response. These phenomena also remind us that although TICT and PICT molecules may have similar structures, they can exhibit different forms of electron transfer, which could impact the judgment in distinguishing between them.

Considering the mechanisms and analyses discussed above, it is worth noting that further efforts are required to enhance predictive performance. The imbalance of TICT and PICT molecules in the dataset can lead to misclassification of the minority class. That means that we need to further expand the dataset for the model. With the continued accumulation of experimental data in the future, increasingly diverse datasets will be incorporated to further iterate and refine our model, thereby enhancing its applicability and robustness. Developing innovative descriptors capable of interpreting the photophysical behavior of fluorescent molecules could better elucidate their underlying mechanisms and ultimately boost the model’s predictive accuracy and specificity.

In conclusion, we propose a novel AI-based approach to deconstructing the structural essence of TICT and PICT without relying on extensive computational costs, thereby facilitating the rational design of corresponding fluorescent probes. We first collected a high-quality dataset of TICT and PICT molecules and established a series of machine learning models for distinguishing between TICT and PICT molecules. The best model achieved an accuracy of 0.784 for the test set. More importantly, benefiting from the model’s excellent interpretability, 5 AI-guided rules based on molecular rigidity and charge transfer driving forces that influence TICT and PICT were uncovered: EDG strength, EWG strength, alkyl cyclization, steric hindrance, and solvent–solute interactions. We also created a more intuitive and practical tool to make it more accessible for researchers to apply in real-world design scenarios. Compared to those designed by community-suggested rules for probe precise design, TICT and PICT fluorophores designed by AI-guided rules achieved obvious improvement; the accuracy of our design increased from 67.47% to 90.96%. This result was fully validated through both quantitative calculations and wet experiments. Therefore, we believe that this work not only effectively deconstructs the structural essence of TICT and PICT but also moves a step closer to the intelligent design of their molecular probes.

## Materials and Methods

### Molecular representation

The properties of TICT and PICT molecules are determined by the combined effects of their 2D and 3D structures and physicochemical properties. In this study, various molecular descriptors and fingerprints were calculated to represent the physicochemical properties of the compounds. The MOE software (version 2018, Chemical Computing Group, Montreal, QC, Canada) was used to calculate 206 2D descriptors. RDKit, CDK, and jCompoundMapper software programs were used to calculate AtomPair, ECFP4, MACCS, CDK, PubChem, DFS, AP3D, and AT3D fingerprints. ChemDes [51] provides detailed information of these fingerprints. Considering the specific properties of TICT and PICT molecules, we also calculated semiempirical calculations on the probe molecules using MOPAC2016 software to obtain their lowest energy conformations, which were used to calculate 3D descriptors by the MOE software. These 3D descriptors were related to potential energy, volume, and conformation-dependent charges.

### Machine learning algorithms

To identify the most suitable machine learning models for TICT and PICT molecules, we employed 12 classical algorithms, including logistic regression, naïve Bayes, DT, radial basis function network, support vector machine, multilayer perceptron, random forest, AdaBoost, gradient boosting classifier, extra trees, XGBoost, LightGBM, and CatBoost. They were implemented in a customized Python (3.8.13) environment equipped with scikit-learn (0.23.2), XGBoost (1.6.2), CatBoost (1.1), LightGBM (3.3.2), and PyCaret (2.3.10). All algorithms were first compared for robustness and predictive performance using PyCaret, after which the selected algorithm was used for model construction and tuning. For all models using classical algorithms, 80% of the randomly partitioned dataset was used for the training set, while the remaining 20% served as the test set to assess model performance. Additionally, a 5-fold CV was applied on the training set to ensure the robustness of the models.

Considering the strong capability of deep learning in identifying nonlinear patterns, MolMapNet was chosen as a representative deep learning method for the comparison [52]. For MolMapNet, the dataset was randomly split into training, validation, and test sets in an 8:1:1 ratio (Fig. S4). The MolMapNet model was built using TensorFlow 2.9.1 on GeForce GTX 1080 Ti.

### Model evaluation metrics

We selected 7 metrics to evaluate the model’s performance: Accuracy (ACC) represents the proportion of correct predictions and reflects the overall accuracy of the model. Precision measures the accuracy of positive predictions, while recall and sensitivity (SE) assess the model’s ability to identify positive samples. Specificity (SP) measures the ability to identify negative samples. The F1 score (F1) is the harmonic mean of precision and recall, while the AUC measures the model’s ability to discriminate between positive and negative samples.

### Feature selection and explanation

For descriptor features, descriptors with a variance less than 0.05 and those with high correlations (greater than 0.95) were removed. Additionally, RFE was further identifying critical impact features on the model. In this process, RFE removes the least important features based on feature importance scores from the model to obtain the feature with the highest accuracy to eliminate redundant features and reduce computational costs (Fig. S2). We applied the SHAP method (SHAP, version 0.41.0) to assess the contribution of individual features. The Shapley values quantify how each feature affects the prediction, and this value possesses an additive property to provide a global overview of feature importance. Crucially, Shapley values reveal the effect direction of each feature in a single prediction to help us attribute prediction errors. In addition, feature importance scores from the model are used to further explore the contribution levels of different features in the model.

### Theoretical calculation

Currently, quantitative calculation is an essential tool for interpreting the emission mechanisms of fluorescent molecules. Especially for fluorescent molecules with dark-state characteristics, quantitative calculation becomes necessary for understanding their properties. Here, we used MOE to perform a conformational search on each compound to obtain the structure with the lowest energy. Then, we optimized the ground-state structure of each compound by Gaussian 16 [53] based on PBE0/6-31g*. Frequency calculations confirmed the absence of imaginary frequencies, indicating that these are indeed the optimal structures. To verify whether these compounds exhibit TICT properties, we performed PES scans of the first excited state for each compound based on TD-PBE0/6-31g* to study their relaxation pathways after excitation. Additionally, we conducted excited-state single-point energy calculations based on TD-PBE0/Def2-TZVP for each compound and employed Multiwfn [54] to perform electron–hole analysis, which investigates their properties upon excitation.

### Chemical experimental characterization

The materials and instruments, detailed synthesis process, and intracellular viscosity imaging methods can be found in the Supporting Materials.

### Spectrometric determination

For spectroscopic measurements, the compound was dissolved in dimethyl sulfoxide to prepare a 10 mM stock solution. Except when noted otherwise, a final concentration of 10 μM was applied in all experiments. In the viscosity detection experiment, methanol and glycerol were mixed in different proportions to obtain solutions with viscosities ranging from 1.24 cP (100% methanol) to 942 cP (100% glycerol). The excitation wavelength was set at 414 nm, and the fluorescence signal was recorded at 554 nm. The slit width was 20 nm for both excitation and emission separately.

### Living cell imaging

Hepa1-6 cells were cultured in 6-well plates for 24 h, followed by incubation with 10 μM compound for 30 min, and then washed 3 times with phosphate-buffered saline buffer (pH = 7.4). Next, the cells were treated with nystatin (10 μM), and the corresponding medium was replaced with fresh medium. The intracellular viscosity was imaged, and fluorescence images of the cells were captured every 30 min using a confocal laser scanning microscope.

## Data Availability

The data that support the findings of this study are available from the corresponding authors upon reasonable request. The code scripts and datasets used in this project are available at https://github.com/ifyoungnet/ChemTICT.

## Supplementary Materials

Supplementary 1 Supplementary materials for structure identification Figs. S1 to S18 Tables S1 to S14

## References

[1] Grabowski ZR, Rotkiewicz K, Rettig W. Structural changes accompanying intramolecular electron transfer: Focus on twisted intramolecular charge-transfer states and structures. Chem Rev. 2003;103(10):3899–4032. 14531716 10.1021/cr940745l

[2] Kuila S, Miranda-Salinas H, Eng J, Li C, Bryce MR, Penfold TJ, Monkman AP. Rigid and planar π-conjugated molecules leading to long-lived intramolecular charge-transfer states exhibiting thermally activated delayed fluorescence. Nat Commun. 2024;15(1):9611. 39511188 10.1038/s41467-024-53740-1 PMC11544105

[3] Li Q, Wu Y, Cao J, Liu Y, Wang Z, Zhu H, Zhang H, Huang F. Pillararene-induced intramolecular through-space charge transfer and single-molecule white-light emission. Angew Chem Int Ed Engl. 2022;61(19): Article e202202381. 35234348 10.1002/anie.202202381

[4] Xing P, Niu Y, Mu R, Wang Z, Xie D, Li H, Dong L, Wang C. A pocket-escaping design to prevent the common interference with near-infrared fluorescent probes in vivo. Nat Commun. 2020;11(1):1573. 32218438 10.1038/s41467-020-15323-8 PMC7099068

[5] Liu X, Qiao Q, Tian W, Liu W, Chen J, Lang MJ, Xu Z. Aziridinyl fluorophores demonstrate bright fluorescence and superior photostability by effectively inhibiting twisted intramolecular charge transfer. J Am Chem Soc. 2016;138(22):6960–6963. 27203847 10.1021/jacs.6b03924

[6] Luo Y, Wang Y, Chen S, Wang N, Qi Y, Zhang X, Yang M, Huang Y, Li M, Yu J, et al. Facile access to twisted intramolecular charge-transfer fluorogens bearing highly pretwisted donor–acceptor systems together with readily fine-tuned charge-transfer characters. Small. 2017;13(20):1604113. 10.1002/smll.201604113 28387442

[7] Zhang W, Kong J, Miao R, Song H, Ma Y, Zhou M, Fang Y. Integrating aggregation induced emission and twisted intramolecular charge transfer via molecular engineering. Adv Funct Mater. 2024;34(7):2311404.

[8] Hanaoka K, Ikeno T, Iwaki S, Deguchi S, Takayama K, Mizuguchi H, Tao F, Kojima N, Ohno H, Sasaki E, et al. A general fluorescence off/on strategy for fluorogenic probes: Steric repulsion-induced twisted intramolecular charge transfer (sr-TICT). Sci Adv. 2024;10: Article eadi8847. 38363840 10.1126/sciadv.adi8847 PMC10871538

[9] Wang C, Chi W, Qiao Q, Tan D, Xu Z, Liu X. Twisted intramolecular charge transfer (TICT) and twists beyond TICT: From mechanisms to rational designs of bright and sensitive fluorophores. Chem Soc Rev. 2021;50(22):12656–12678. 34633008 10.1039/d1cs00239b

[10] Lou AJ-T, Righetto S, Barger C, Zuccaccia C, Cariati E, Macchioni A, Marks TJ. Unprecedented large hyperpolarizability of twisted chromophores in polar media. J Am Chem Soc. 2018;140(28):8746–8755. 29909629 10.1021/jacs.8b04320

[11] Dong H, Wei Y, Zhang W, Wei C, Zhang C, Yao J, Zhao YS. Broadband tunable microlasers based on controlled intramolecular charge-transfer process in organic supramolecular microcrystals. J Am Chem Soc. 2016;138(4):1118–1121. 26756966 10.1021/jacs.5b11525

[12] Fischer T, Leitner J, Gerwien A, Mayer P, Dreuw A, Dube H, Wachtveitl J. Mechanistic elucidation of the Hula-Twist photoreaction in hemithioindigo. J Am Chem Soc. 2023;145(27):14811–14822. 37364887 10.1021/jacs.3c03536 PMC10347542

[13] Xia B, Ren F, Ma X, Yang ZC, Jiang Z, Fang W, Wang N, Hu J, Zhu W, He T, et al. Preparation of NIR-II polymer nanoprobe through twisted intramolecular charge transfer and Förster resonance energy transfer of NIR-I dye. Adv Healthc Mater. 2024;13(22):e2400760. 38703026 10.1002/adhm.202400760

[14] Song Y, Liang C. H-bond engineering as a general strategy for inhibiting twisted intramolecular charge transfer in donor–acceptor fluorescent probes: Reshaping the pre-twisting method. Talanta. 2024;272: Article 125770. 38340393 10.1016/j.talanta.2024.125770

[15] Huang Y, Zeng X, Ma X, Lin Z, Sun J, Xiao W, Liu SH, Yin J, Yang G. A visible light-activated azo-fluorescent switch for imaging-guided and light-controlled release of antimycotics. Nat Commun. 2024;15(1):8670. 39375340 10.1038/s41467-024-52855-9 PMC11458760

[16] Wang G, Wan Z, Cai Z, Li J, Li Y, Hu X, Lei D, Dou X. Complete inhibition of the rotation in a barrierless TICT probe for fluorescence-on qualitative analysis. Anal Chem. 2022;94(33):11679–11687. 35948453 10.1021/acs.analchem.2c02407

[17] Zhang Z, Gou Z, Dong B, Tian M. Tuning the “critical polarity” of TICT dyes: Construction of polarity-sensitive platform to distinguish duple organelles. Sens Actuators B Chem. 2022;355: Article 131349.

[18] Sasaki S, Suzuki S, Sameera WMC, Igawa K, Morokuma K, Konishi G. Highly twisted *N*,*N*-dialkylamines as a design strategy to tune simple aromatic hydrocarbons as steric environment-sensitive fluorophores. J Am Chem Soc. 2016;138(26):8194–8206. 27300152 10.1021/jacs.6b03749

[19] Naito H, Nishino K, Morisaki Y, Tanaka K, Chujo Y. Solid-state emission of the anthracene-*o*-carborane dyad from the twisted-intramolecular charge transfer in the crystalline state. Angew Chem Int Ed Engl. 2017;56(1):254–259. 27911472 10.1002/anie.201609656

[20] Ye Z, Yang W, Wang C, Zheng Y, Chi W, Liu X, Huang Z, Li X, Xiao Y. Quaternary piperazine-substituted rhodamines with enhanced brightness for super-resolution imaging. J Am Chem Soc. 2019;141(37):14491–14495. 31487156 10.1021/jacs.9b04893

[21] Miao R, Li J, Wang C, Jiang X, Gao Y, Liu X, Wang D, Li X, Liu X, Fang Y. A general method to develop highly environmentally sensitive fluorescent probes and AIEgens. Adv Sci. 2022;9(5):2104609. 10.1002/advs.202104609 PMC8844555 34927375

[22] Liu X, Cho B, Chan L, Kwan WL, Lee C. Development of asymmetrical near-infrared squaraines with large Stokes shift. RSC Adv. 2015;5:106868–106876.

[23] Lum K, Zielinski SM, Abelt CJ. Dansyl emits from a PICT excited state. J Phys Chem A. 2021;125(5):1229–1233. 33528253 10.1021/acs.jpca.0c11229

[24] Liu Z, Lavis LD, Betzig E. Imaging live-cell dynamics and structure at the single-molecule level. Mol Cell. 2015;58(4):644–659. 26000849 10.1016/j.molcel.2015.02.033

[25] Cao J, Liu Q, Bai S, Wang H, Ren X, Xu Y. Ladder-type dye with large transition dipole moment for solvatochromism and microphase visualization. ACS Appl Mater Interfaces. 2019;11(33):29814–29820. 31340645 10.1021/acsami.9b07677

[26] Debnath S, Mohanty A, Naik P, Salzner U, Dasgupta J, Patil S. Deciphering intramolecular charge transfer in fluoranthene derivatives. J Mater Chem C. 2024;12:9200–9209.

[27] Yoshihara T, Druzhinin SI, Zachariasse KA. Fast intramolecular charge transfer with a planar rigidized electron donor/acceptor molecule. J Am Chem Soc. 2024;126:8535–8539. 10.1021/ja049809s 15238011

[28] Parusel ABJ. Excited state intramolecular charge transfer in *N*,*N*-heterocyclic-4-aminobenzonitriles: A DFT study. Chem Phys Lett. 2001;340(5–6):531–537.

[29] Zhang Z, Zhang G, Wang J, Sun S. The mechanisms of Large Stokes Shift and Fluorescence Quantum Yields in anilino substituted Rhodamine analogue: TICT and PICT. Comput Theor Chem. 2016;1095:44–53.

[30] Han F, Abedi S, He S, Zhang H, Long S, Zhou X, Chanmungkalakul S, Ma H, Sun W, Liu X, et al. Aryl-modified pentamethyl cyanine dyes at the C2′ position: A tunable platform for activatable photosensitizers. Adv Sci. 2024;11(7):2305761. 10.1002/advs.202305761 PMC10870032 38063803

[31] Wang L, Li N, Wang W, Mei A, Shao J, Wang W, Dong X. Benzobisthiadiazole-based small molecular near-infrared-II fluorophores: From molecular engineering to nanophototheranostics. ACS Nano. 2024;18(6):4683–4703. 38295152 10.1021/acsnano.3c12316

[32] Lei Z, Zhang F. Molecular engineering of NIR-II fluorophores for improved biomedical detection. Angew Chem Int Ed Engl. 2021;60(30):16294–16308. 32780466 10.1002/anie.202007040

[33] Jödicke C, Lüthi HP. Time-dependent density functional theory (TDDFT) study of the excited charge-transfer state formation of a series of aromatic donor–acceptor systems. J Am Chem Soc. 2003;125(1):252–264. 12515528 10.1021/ja020361+

[34] Wang C, Qiao Q, Chi W, Chen J, Liu W, Tan D, McKechnie S, Lyu D, Jiang X-F, Zhou W, et al. Quantitative design of bright fluorophores and AIEgens by the accurate prediction of twisted intramolecular charge transfer (TICT). Angew Chem Int Ed Engl. 2020;59(25):10160–10172. 31943591 10.1002/anie.201916357

[35] Zhong C. The driving forces for twisted or planar intramolecular charge transfer. Phys Chem Chem Phys. 2015;17:9248–9257. 25759940 10.1039/c4cp02381a

[36] Olivier Y, Sancho-Garcia J, Muccioli L, D’Avino G, Beljonne D. Computational design of thermally activated delayed fluorescence materials: The challenges ahead. J Phys Chem Lett. 2018;9(20):6149–6163. 30265539 10.1021/acs.jpclett.8b02327

[37] Gieseking RL, Mukhopadhyay S, Risko C, Marder SR, Brédas J-L. 25th anniversary article: Design of polymethine dyes for all-optical switching applications: Guidance from theoretical and computational studies. Adv Mater. 2014;26(1):68–84. 24302357 10.1002/adma.201302676

[38] Zhenkun S, Rui D, Qianqian Y, Zhitao M, Ruoyu W, Haoran L, Xiaoping L, Hongwu M. Enzyme commission number prediction and benchmarking with hierarchical dual-core multitask learning framework. Research. 2023;6:0153. 37275124 10.34133/research.0153 PMC10232324

[39] Qi R, Zou Q. Trends and potential of machine learning and deep learning in drug study at single-cell level. Research. 2023;6: Article 0050. 36930772 10.34133/research.0050 PMC10013796

[40] Qiu J, Wang K, Lian Z, Yang X, Huang W, Qin A, Wang Q, Tian J, Tang B, Zhang S. Prediction and understanding of AIE effect by quantum mechanics-aided machine-learning algorithm. Chem Commun. 2018;54:7955. 10.1039/c8cc02850h 29956696

[41] Dong J, Qian J, Yu K, Huang S, Cheng X, Chen F, Jiang H, Zeng W. Rational design of organelle-targeted fluorescent probes: Insights from artificial intelligence. Research. 2023;6:0075. 36930810 10.34133/research.0075 PMC10013958

[42] Zhu Y, Fang Y, Huang W, Zhang W, Chen F, Dong J, Zeng W. AI-driven precision subcellular navigation with fluorescent probes. J Mater Chem B. 2024;12:11054–11062. 39392117 10.1039/d4tb01835d

[43] Sumita M, Terayama K, Suzuki N, Ishihara S, Tamura R, Chahal MK, Payne DT, Yoshizoe K, Tsuda K. De novo creation of a naked eye–detectable fluorescent molecule based on quantum chemical computation and machine learning. Sci Adv. 2022;8(10):eabj3906. 35263133 10.1126/sciadv.abj3906 PMC8906732

[44] Dong J, Yao Z, Zhang L, Luo F, Lin Q, Lu A, Chen AF, Cao D. PyBioMed: A Python library for various molecular representations of chemicals, proteins and DNAs and their interactions. J Cheminform. 2018;10:16. 29556758 10.1186/s13321-018-0270-2 PMC5861255

[45] Dong J, Wang N, Yao Z, Zhang L, Cheng Y, Ouyang D, Lu A, Cao D. ADMETlab: A platform for systematic ADMET evaluation based on a comprehensively collected ADMET database. J Cheminform. 2018;10:1. 29943074 10.1186/s13321-018-0283-x PMC6020094

[46] Xiong G, Wu Z, Yi J, Fu L, Yang Z, Hsieh C, Yin M, Zeng X, Wu C, Lu A, et al. ADMETlab 2.0: An integrated online platform for accurate and comprehensive predictions of ADMET properties. Nucleic Acids Res. 2021;49(W1):W5–W14. 33893803 10.1093/nar/gkab255 PMC8262709

[47] Li L, Li K, Li M, Shi L, Liu Y, Zhang H, Pan S, Wang N, Zhou Q, Yu X. BODIPY-based two-photon fluorescent probe for real-time monitoring of lysosomal viscosity with fluorescence lifetime imaging microscopy. Anal Chem. 2018;90(9):5873–5878. 29600703 10.1021/acs.analchem.8b00590

[48] Wang H, Sun Y, Lin X, Feng W, Li Z, Yu M. Multi-organelle-targeting pH-dependent NIR fluorescent probe for lysosomal viscosity. Chin Chem Lett. 2022;34(3): Article 107626.

[49] Zhang T, Huo F, Yin C. TICT improved NIR emission and lysosome-specific functional dye visualizing viscosity in disease model. Sens Actuators B Chem. 2023;404: Article 135236.

[50] Kopansky-Groisman E, Kogan-Zviagin I, Sella-Tavor O, Oron-Herman M, David A. Near-infrared fluorescent activated polymeric probe for imaging intraluminal colorectal cancer tumors. Biomacromolecules. 2019;20(9):3547–3556. 31381303 10.1021/acs.biomac.9b00806

[51] Dong J, Cao D, Miao H-Y, Liu S, Deng B, Yun Y, Wang N, Lu A-P, Zeng W, Chen AF. ChemDes: An integrated web-based platform for molecular descriptor and fingerprint computation. J Cheminform. 2015;7:60. 26664458 10.1186/s13321-015-0109-z PMC4674923

[52] Shen W, Zeng X, Zhu F, Wang Y, Qin C, Tan Y, Jiang Y, Chen YZ. Out-of-the-box deep learning prediction of pharmaceutical properties by broadly learned knowledge-based molecular representations. Nat Mach Intell. 2021;3:334–343.

[53] Frisch MJ, Trucks GW, Schlegel HB, Scuseria GE, Robb MA, Cheeseman JR, Scalmani G, Barone V, Petersson GA, Nakatsuji H, et al. Gaussian 16, revision A.03. Gaussian, Inc.: Wallingford, CT; 2016.

[54] Lu T, Chen F. Multiwfn: A multifunctional wavefunction analyzer. J Comput Chem. 2012;33(5):580–592. 22162017 10.1002/jcc.22885

