# Supplementary material for: Interpretable Artificial Intelligence Decodes the Chemical Structural Essence of Twisted Intramolecular Charge Transfer and Planar Intramolecular Charge Transfer Fluorophores
Source: Research (Wash D C). 2025 Dec 9;8:1021. doi: 10.34133/research.1021 (PMC12686344; doi:10.34133/research.1021)

Supporting Information

Supplementary materials for structure identification

Materials and instruments

Unless otherwise stated, all chemicals with required purities were purchased from Energy Chemical Co., Ltd (Shanghai, China). ^{1}H-NMR and ^{13}C-NMR spectra were recorded on Bruker AVANCEIII 500M spectrometer (Rheinstetten, Germany) and Bruker AVANCEIII 700M spectrometer (Rheinstetten, Germany). High-resolution mass spectroscopy (HRMS) was obtained on an Orbitrap Velos Pro LC-MS spectrometer (Thermo Scientific). UV–vis absorption spectra were measured on a Shimadzu UV-2450 UV-VIS spectrophotometer. Fluorescence spectra were measured with a HITACHI F-2700 fluorescence spectrophotometer (HITACHI, Japan). Fluorescence images of cells were acquired on a Leica TCS SP8 laser scanning confocal microscope (Germany).

Compound synthesis

Synthetic route of Compound 1

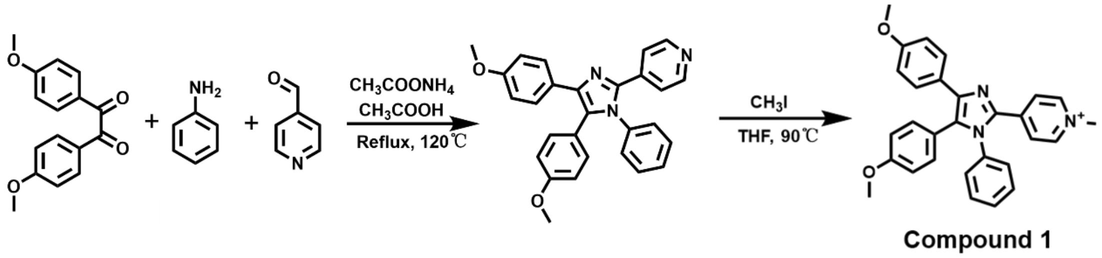

Compound 1

Aniline (0.091 mL, 1.00 mmol) and 4-pyridine carboxaldehyde (0.106 g, 1.00 mmol) and were dissolved in acetic acid (10 mL) and stirred for 1 h at room temperature. Benzil (0.210 g, 1.00 mmol) and ammonium acetate (0.355 g, 4.60 mmol) were added subsequently. The mixture was heated at 120 °C overnight. After quench of the reaction, the dark solution was poured into copious amounts of water. After neutralization, the mixture was filtered and washed with water. Reprecipitation in methanol from dichloromethane solution afforded a white powder (0.157 g, yield = 42%). White powder (0.19 g, 0.50 mmol) in 25 mL THF was reflux with Methyl iodide (0.071 g, 0.50 mmol) for 8 hrs. After the mixture cooled to room temperature, it was evaporated under reduced pressure to remove the solvents. The residue was purified by a silica gel column chromatography using a DCM and methanol mixture (50:1, v/v) as the elution solvent to give compound 1 as a green powder (87.3 mg, yield: 45%).^{1}H NMR (700 MHz, Chloroform-*d*) δ 8.89 (d, J = 6.6 Hz, 2H), 7.79 (d, J = 6.5 Hz, 2H), 7.53 – 7.49 (m, 3H), 7.47 (t, J = 7.5 Hz, 2H), 7.21 – 7.18 (m, 2H), 7.04 (d, J = 8.3 Hz, 2H), 6.84 – 6.81 (m, 2H), 6.79 – 6.75 (m, 2H), 4.54 (s, 3H), 3.78 (dd, J = 16.3, 1.9 Hz, 6H). ^{13}C NMR (176 MHz, $CDCl_3$) δ 159.99, 159.28, 144.52, 144.34, 141.79, 139.22, 136.09, 135.61, 132.08, 130.55, 130.53, 128.56, 127.98, 125.55, 123.44, 120.56,

114.21, 113.89, 55.31, 55.23, 48.56. HR-MS (m/z): calculated for $C_{29}H_{26}N_3O_2^+$ [M+H]$^+$, 449.2054; found, 449.2054.

Synthetic route of Compound 2

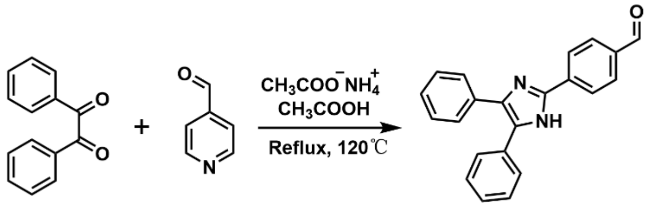

Compound 2

Compound 2 was prepared according to the reported reference[1]. Benzil (210 mg, 1 mmol), 1,4-phthalaldehyde (134 mg, 5 mmol) and ammonium acetate (620 mg，8 mmol) were dissolved in acetic acid, and reacted at 110 °C for 6 h with an inert atmosphere of nitrogen. After completely reacted, the reaction solution was cooled to room temperature, poured into ice water, suction filtered, washed with water and dried in vacuo. The resulting residue was purified by column chromatography on silica gel (petroleum ether to ethyl acetate/petroleum ether =1:10, v/v) to afford the Compound 2 as a yellow powder (276 mg, yield: 85%).

References

1. Liu, S.; Yang, M.; Liu, Y.; Chen, H.; Li, H. A novel "turn-on" fluorescent probe based on triphenylimidazole-hemicyanine dyad for colorimetric detection of CN^- in 100% aqueous solution. *J. Hazard. Mater.* **344**, 875–882 (2018).

Tables

Table S1. The number of molecules for three types of 3D descriptors.

Set	Method	Sum	TICT	PICT
Modeling	AM1	863	479	384
	PM3	873	487	386
	MNDO	868	488	380

Table S2. Model results of descriptors and molecular fingerprints combined with algorithms.

Feature	Model	CV					Test						
		ACC	AUC	Recall	Prec.	F1	Acc	AUC	Recall	Prec.	SE	SP	F1
2D	CatBoost	0.709	0.758	0.819	0.725	0.768	0.710	0.783	0.796	0.716	0.796	0.603	0.754
MACCS	CatBoost	0.713	0.753	0.815	0.723	0.766	0.733	0.757	0.829	0.750	0.829	0.592	0.787
ECFP4	Extra Trees	0.713	0.767	0.773	0.742	0.756	0.761	0.806	0.800	0.800	0.800	0.704	0.800
AtomPair	XGBoost	0.722	0.753	0.803	0.738	0.768	0.784	0.846	0.771	0.711	0.771	0.792	0.740
CDK	CatBoost	0.720	0.788	0.838	0.728	0.779	0.733	0.785	0.636	0.721	0.636	0.808	0.676
Pubchem	Lightgbm	0.697	0.746	0.765	0.733	0.748	0.716	0.762	0.765	0.735	0.765	0.654	0.750
jCompoundMapper_DFS	CatBoost	0.722	0.764	0.831	0.720	0.771	0.705	0.769	0.798	0.758	0.798	0.532	0.778
jCompoundMapper_AP3D	ExtraTrees	0.727	0.785	0.851	0.732	0.787	0.710	0.767	0.811	0.700	0.811	0.593	0.751
jCompoundMapper_AT3D	CatBoost	0.715	0.775	0.846	0.710	0.772	0.767	0.818	0.843	0.791	0.843	0.647	0.816

Table S3. Model results of 2D+3D descriptors and molecular fingerprints combined with algorithms.

Characterization		ACC_cv	ACC_test	RFE		Model	CV					Test						
		mean ± std	mean ± std	Optimal number of features	Optimal accuracy		Acc	AUC	Recall	Prec.	F1	Acc	AUC	Recall	Prec.	SE	SP	F1
2D+3D	AM1	0.715±0.014	0.717±0.032	104	0.654	CatBoost Classifier	0.725	0.785	0.788	0.731	0.758	0.734	0.757	0.822	0.748	0.822	0.611	0.783
	PM3	0.712±0.014	0.711±0.029	78	0.660	Extra Trees Classifier	0.733	0.798	0.825	0.734	0.776	0.734	0.787	0.804	0.725	0.804	0.654	0.763
	MNDO	0.709±0.013	0.712±0.04	130	0.658	CatBoost Classifier	0.714	0.776	0.805	0.714	0.756	0.738	0.796	0.784	0.777	0.784	0.671	0.780

Table S4. Model results of deep learning (MolMapNet).

Characterization	Best epoch performance					Test						
	loss	$loss_{val}$	auc	auc_{val}	epoch	Acc	AUC	Recall	Prec.	SE	SP	F1
Dual	0.137	0.482	0.999	0.834	35	0.716	0.778	0.736	0.780	0.736	0.686	0.757
Descriptor	0.382	0.561	0.934	0.778	97	0.761	0.804	0.759	0.837	0.759	0.765	0.796
Fingerprint	0.287	0.566	0.992	0.771	23	0.773	0.826	0.778	0.840	0.778	0.765	0.808

Table S5. The permutation importance of 2D descriptors.

NO	Feature	Importance	Model_based rank	SHAP	SHAP_based rank	Class	Description
6	GCUT_SLOGP_0	8.04	1	70.96	1	2D	LogP GCUT (0/3)
44	SMR_VSA7	3.49	2	24.00	2	2D	Bin 7 SMR (0.560,10]
16	Kier3	3.06	3	18.89	4	2D	Third kappa shape index
24	PEOE_VSA+2	3.06	4	14.42	8	2D	Total positive 2 vdw surface area
31	rsynth	2.94	5	9.57	16	2D	Synthetic Feasibility
25	PEOE_VSA-0	2.92	6	15.92	5	2D	Total negative 0 vdw surface area
26	PEOE_VSA_NEG	2.86	7	8.72	23	2D	Total negative vdw surface area
3	balabanJ	2.59	8	12.78	10	2D	Balaban averaged distance sum connectivity
14	h_pstates	2.54	9	14.75	6	2D	Entropic state count (pH=7)
37	SlogP_VSA9	2.19	10	6.89	30	2D	Bin 9 SlogP (0.40,10]
42	SMR_VSA4	2.17	11	11.03	12	2D	Bin 4 SMR (0.390,0.440]
11	h_log_pbo	2.16	12	10.73	13	2D	Sum of log (1 + p-bond orders)
32	SlogP_VSA0	2.16	13	10.38	14	2D	Bin 0 SlogP (-10 ,-0.40]
8	h_emd	2.08	14	9.56	17	2D	Sum of EHT donor strengths
48	vsa_pol	2.07	15	9.07	20	2D	VDW polar surface area (A**2)

Table S6. The permutation importance of 2D+3D descriptors (AM1).

NO	Feature	Importance	Model_based rank	SHAP	SHAP_based rank	Class	Description
61	GCUT_SLOGP_0	3.50	1	35.46	1	2D	LogP GCUT (0/3)
33	vsurf_CW2	3.42	2	29.29	2	i3D	Capacity factor at -0.5
93	SlogP_VSA0	2.86	3	21.67	4	2D	Bin 0 SlogP (-10 ,-0.40]
85	Q_VSA_HYD	2.25	4	24.26	3	2D	Total hydrophobic vdw surface area
37	vsurf_DD23	1.85	5	16.62	5	i3D	vsurf_EDmin2, vsurf_EDmin3 distance
94	SlogP_VSA5	1.76	6	12.85	7	2D	Bin 5 SlogP (0.15, 0.20]
78	PEOE_VSA+0	1.72	7	10.01	13	2D	Total positive 0 vdw surface area
99	SMR_VSA7	1.66	8	15.34	6	2D	Bin 7 SMR (0.560,10]
34	vsurf_CW3	1.63	9	7.78	18	i3D	Capacity factor at -1.0
70	Kier3	1.59	10	10.53	11	2D	Third kappa shape index
80	PEOE_VSA-0	1.55	11	10.86	10	2D	Total negative 0 vdw surface area
89	Q_VSA_POS	1.50	12	11.64	9	2D	Total positive vdw surface area
13	dipoleZ	1.48	13	9.95	14	x3D	Dipole moment (Z)
0	AM1_dipole	1.46	14	7.82	17	i3D	Dipole moment
17	E_oop	1.45	15	6.73	26	i3D	Out-of-plane Energy

Table S7. The permutation importance of 2D+3D descriptors (PM3).

NO	Feature	Importance	Model_based rank	SHAP	SHAP_based rank	Class	Description
69	SlogP_VSA5	0.0260	1	3.37	1	2D	Bin 5 SlogP (0.15, 0.20)
28	vsurf_CW2	0.0255	2	2.99	2	i3D	Capacity factor at -0.5
66	Q_VSA_POS	0.0192	3	2.07	3	2D	Total positive vdw surface area
29	vsurf_CW3	0.0177	4	1.73	5	i3D	Capacity factor at -1.0
70	SMR_VSA7	0.0176	5	1.74	4	2D	Bin 7 SMR (0.560,10]
67	rsynth	0.0165	6	1.65	6	2D	Synthetic Feasibility
60	PEOE_VSA-0	0.0162	7	1.47	7	2D	Total negative 0 vdw surface area
58	PEOE_VSA+0	0.0156	8	1.28	11	2D	Total positive 0 vdw surface area
75	E_vdw	0.0153	9	1.29	10	i3D	Van der Waals energy
38	vsurf_IW1	0.0149	10	1.32	8	i3D	Hydrophilic integy moment at -0.2
42	vsurf_IW6	0.0148	11	0.69	39	i3D	Hydrophilic integy moment at -4.0
49	GCUT_SLOGP_0	0.0148	12	1.31	9	2D	LogP GCUT (0/3)
74	E_strain	0.0142	13	0.95	23	i3D	E minus energy of local minimum
32	vsurf_HB1	0.0141	14	1.10	17	i3D	H-bond donor capacity at -0.2
51	h_emd_C	0.0141	15	1.07	18	2D	Sum of EHT carbon donor strengths

Table S8. The permutation importance of 2D+3D descriptors (MNDO).

NO	Feature	Importance	Model_based rank	SHAP	SHAP_based rank	Class	Description
64	GCUT_SLOGP_0	3.72	1	31.97	1	2D	LogP GCUT (0/3)
32	vsurf_CW2	2.99	2	24.94	3	i3D	Capacity factor at -0.5
93	Q_VSA_HYD	2.46	3	25.93	2	2D	Total hydrophobic vdw surface area
48	vsurf_IW2	2.35	4	10.78	7	i3D	Hydrophilic integy moment at -0.5
3	AM1_LUMO	2.33	5	13.54	4	i3D	LUMO energy (eV)
19	E_tor	1.80	6	12.11	5	i3D	Torsion energy
100	SlogP_VSA0	1.67	7	11.69	6	2D	Bin 0 SlogP (-10 ,-0.40]
0	AM1_dipole	1.50	8	6.68	17	i3D	Dipole moment
47	vsurf_IW1	1.47	9	10.18	8	i3D	Hydrophilic integy moment at -0.2
15	E_ang	1.38	10	4.64	31	i3D	Angle Bend Energy
85	PEOE_VSA+3	1.36	11	5.77	19	2D	Total positive 3 vdw surface area
104	SlogP_VSA5	1.32	12	9.66	10	2D	Bin 5 SlogP (0.15, 0.20]
41	vsurf_HB1	1.26	13	8.84	12	i3D	H-bond donor capacity at -0.2
34	vsurf_D8	1.23	14	5.96	18	i3D	Hydrophobic volume at -1.6
74	Kier3	1.20	15	9.72	9	2D	Third kappa shape index

Table S9. The permutation importance of MACCS fingerprint.

NO	Feature	Importance	Model_rank	SHAP	SHAP_rank	Description
150	MACCS150	4.0901	1	38.03939	1	('*!@*@*!@*',0), # A!A$A!A
154	MACCS154	3.9922	2	33.02807	2	('[#6]=[#8]',0), # C=O
139	MACCS139	2.8137	3	25.90223	3	('[O;!H0]',0), # OH
133	MACCS133	2.6411	4	20.55539	4	('*@*!@[#7]',0), # A$A!N
164	MACCS164	2.4586	5	19.52402	5	('[#8]',0), # O
105	MACCS105	2.0789	8	17.65287	6	('*@*(@*)@*',0), # A$A($A)$A
101	MACCS101	1.8106	12	15.77741	10	# 8M Ring or larger. This only handles up to ring sizes of 14
119	MACCS119	2.0181	10	14.26748	11	('[#7]=*',0), # N=A
116	MACCS116	2.2228	6	13.45542	12	('[$([CH3]~*~*~[CH2]~*),$([CH3]~*1~*~[CH2]1)]',0), # CH3AACH2A
57	MACCS57	2.0393	9	13.05583	13	('[#8R]',0), # O Heterocycle
80	MACCS80	1.7512	14	11.86087	15	('[#7]~*~*~*~[#7]',0), # NAAAN

Table S10. Permutation feature importance by descriptor model (MolMapNet)

Subtypes	Top 50	Top 500	percentage of 50	percentage of 500	Total descriptors
Autocorr	15	238	30.00%	47.60%	597
Charge	1	13	2.00%	2.60%	25
Connectivity	1	7	2.00%	1.40%	53
Constitution	0	15	0.00%	3.00%	63
Estate	19	110	38.00%	22.00%	220
Fragment	1	9	2.00%	1.80%	84
InfoContent	1	21	2.00%	4.20%	42
Kappa	0	0	0.00%	0.00%	8
Matrix	0	37	0.00%	7.40%	141
MOE	0	12	0.00%	2.40%	51
Path	4	8	8.00%	1.60%	18
Property	4	11	8.00%	2.20%	18
Topology	4	19	8.00%	3.80%	24
NaN	0	0	0.00%	0.00%	25

Table S11. Permutation Feature Importance by fingerprint model (MolMapNet)

Subtypes	Top 50	Top 500	percentage of 100	percentage of 500	Total bits
MACCSFP	33	94	33.00%	18.80%	162
PharmacoErGFP	6	122	6.00%	24.40%	421
PubChemFP	61	284	61.00%	56.80%	720

Table S12. Single descriptor channel model interpretation of MolMapNet.

**Descriptor name**	**Feature importance based on test set**	**Subtypes**	**Description**
MINaasN	0.023396238	Estate	Minimum atom-type E-State: :N:-
MAXsssN	0.017712994	Estate	Maximum atom-type E-State: >N-
MolQedWeightsNone	0.016575913	Property	QED MolDs Quantitative estimation
MAXaasN	0.015658377	Estate	Maximum atom-type E-State: :N:-
MINsssN	0.014543081	Estate	Minimum atom-type E-State: >N-
GATS1i	0.011825845	Autocorr	Geary autocorrelation - lag 1 / weighted by first ionization potential
MAXaasC	0.009950234	Estate	Maximum atom-type E-State: :C:-
NChargeMean	0.00934645	Charge	
UniqueSubgraphsOfLength1	0.007917705	Path	
MAXddssS	0.007248105	Estate	Maximum atom-type E-State: >S==
MINdsCH	0.007113359	Estate	Minimum atom-type E-State: =CH-
MINssssB	0.007030992	Estate	Minimum atom-type E-State: >B<-
MAXssssB	0.006853679	Estate	Maximum atom-type E-State: >B<-
MAXaaCH	0.006514181	Estate	Maximum atom-type E-State: :CH:
MAXdsCH	0.006179613	Estate	Maximum atom-type E-State: =CH-
AATS1i	0.006152018	Autocorr	Average Broto-Moreau autocorrelation - lag 1 / weighted by first ionization potential
MINddssS	0.006150987	Estate	Minimum atom-type E-State: >S==
IC4	0.005844576	InfoContent	-
AATS4i	0.005698075	Autocorr	Average Broto-Moreau autocorrelation - lag 4 / weighted by first ionization potential
AATS2i	0.00561766	Autocorr	Average Broto-Moreau autocorrelation - lag 2 / weighted by first ionization potential

Table S13. Single molecular fingerprint channel model interpretation of MolMapNet.

**Descriptor name**	**Feature importance based on test set**	**Subtypes**	**Bit Substructure**
PharmacoErGFP123	0.003839033	PharmacoErGFP	('Donor', 'Aromatic', 19)
MACCSFP158	0.003378408	MACCSFP	('[#6]-[#7]', 0)
MACCSFP148	0.002998571	MACCSFP	('*~[!#6;!#1](~*)~*', 0)
PharmacoErGFP124	0.002997089	PharmacoErGFP	('Donor', 'Aromatic', 20)
MACCSFP150	0.002870144	MACCSFP	('*!@*@*!@*', 0)
MACCSFP133	0.002749117	MACCSFP	('*@*!@[#7]', 0)
PharmacoErGFP404	0.002564137	PharmacoErGFP	('Hydrophobic', 'Aromatic', 6)
MACCSFP122	0.002489018	MACCSFP	('*~[#7](~*)~*', 0)
MACCSFP149	0.002420051	MACCSFP	('[C;H3,H4]', 1)
PubChemFP656	0.002286637	PubChemFP	C-C-N-C-C
PubChemFP344	0.002282785	PubChemFP	C(~C)(~H)
MACCSFP156	0.002280827	MACCSFP	('[#7]~*(~*)~*', 0)
PharmacoErGFP125	0.002167669	PharmacoErGFP	('Donor', 'Aromatic', 21)
PubChemFP660	0.002106939	PubChemFP	C-C=C-C-C
MACCSFP165	0.002095358	MACCSFP	('[R]', 0)
PubChemFP356	0.002075674	PubChemFP	C(~C)(:C)(:C)
PubChemFP585	0.001959768	PubChemFP	C-C:C-N-C
PubChemFP600	0.00193418	PubChemFP	N-C:C:C-C
PubChemFP385	-0.001929696	PubChemFP	C(:C)(:C)(:C)
PubChemFP2	0.001893443	PubChemFP	>= 16 H

Table S14. The absorption and fluorescence spectra of Compound 1 and Compound 2 in different solvents.

Compound		EA	ACN	THF	DMSO	MeOH	DCM	Gly
Compound 1	λ_{abs}(nm)	411	414	410	410	417	450	414
	λ_{Flu} (nm)	-	-	-	-	-	-	514
Compound 2	λ_{abs}(nm)	361	359	365	368	355	365	364
	λ_{Flu} (nm)	471	499	461	493	522	496	521

Figures

Fig. S1. The principal chemical scaffolds of the 574 TICT and 414 PICT molecules.

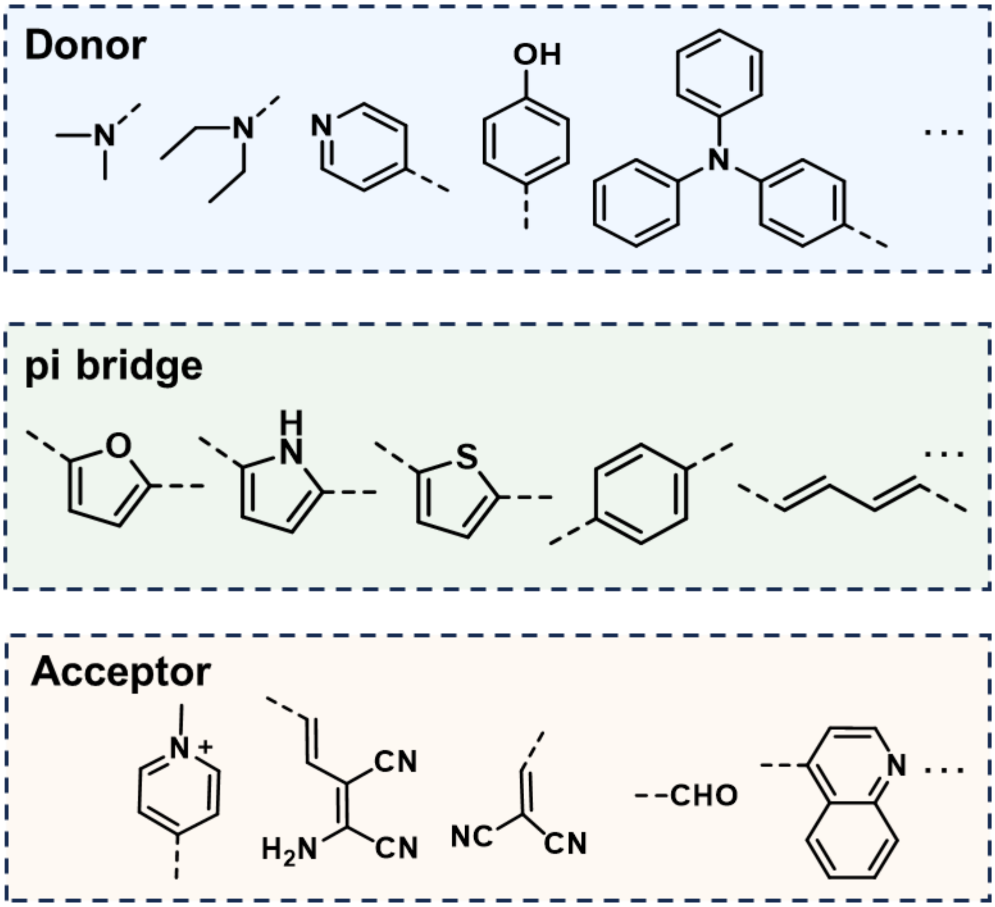

Fig. S2. Feature selection based on RFE.

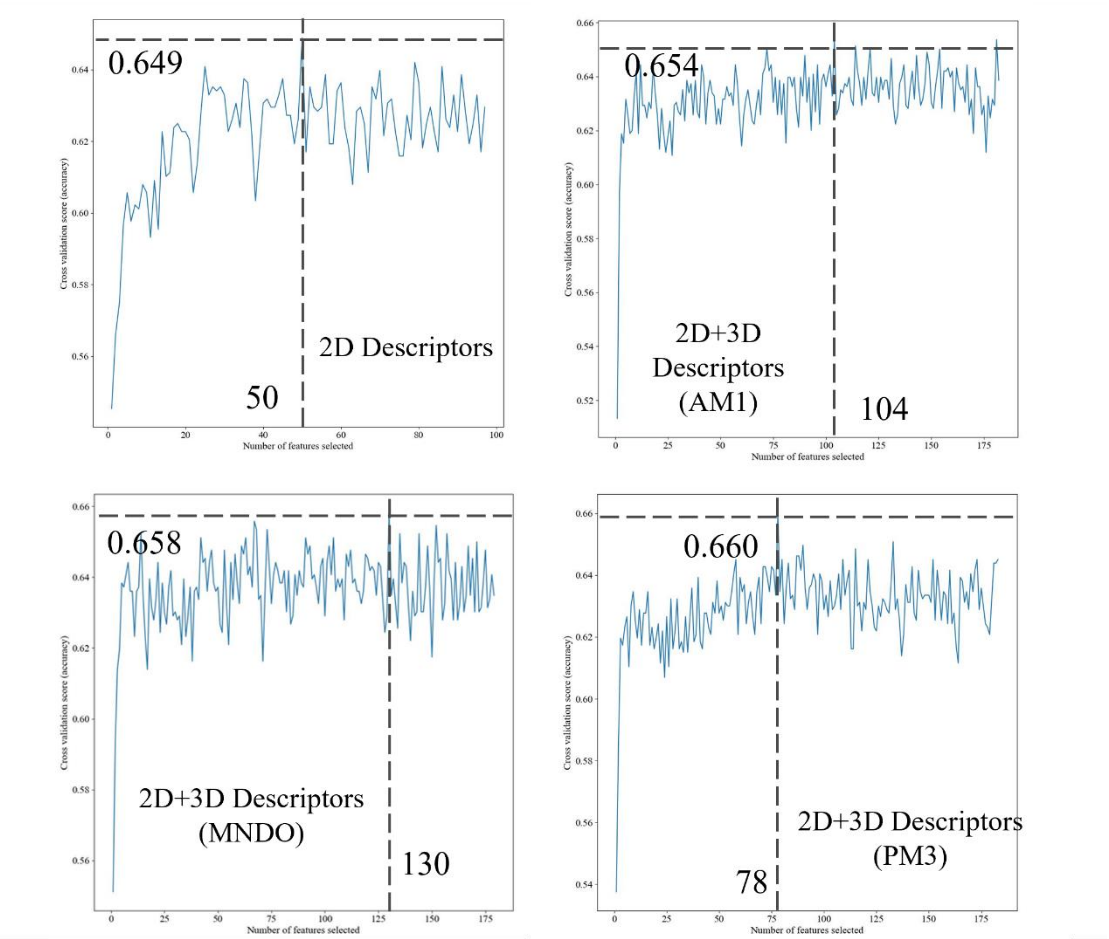

Fig. S3. Optimal model test set classification results.

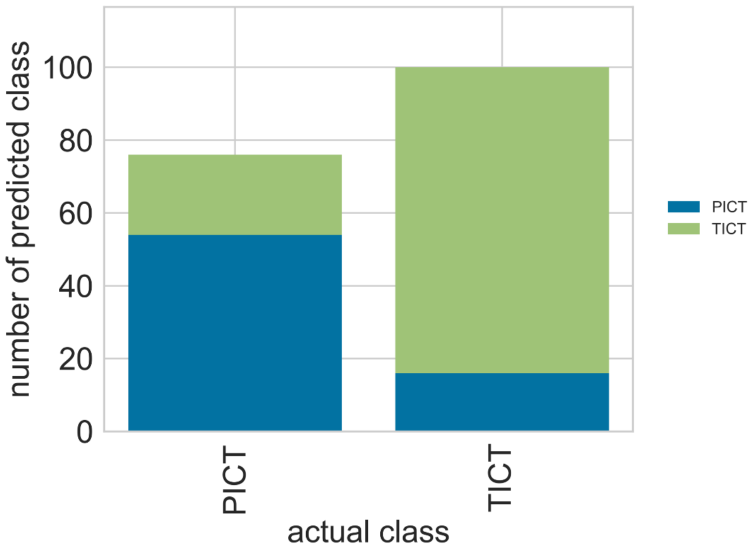

Fig. S4. The framework of MolMapNet.

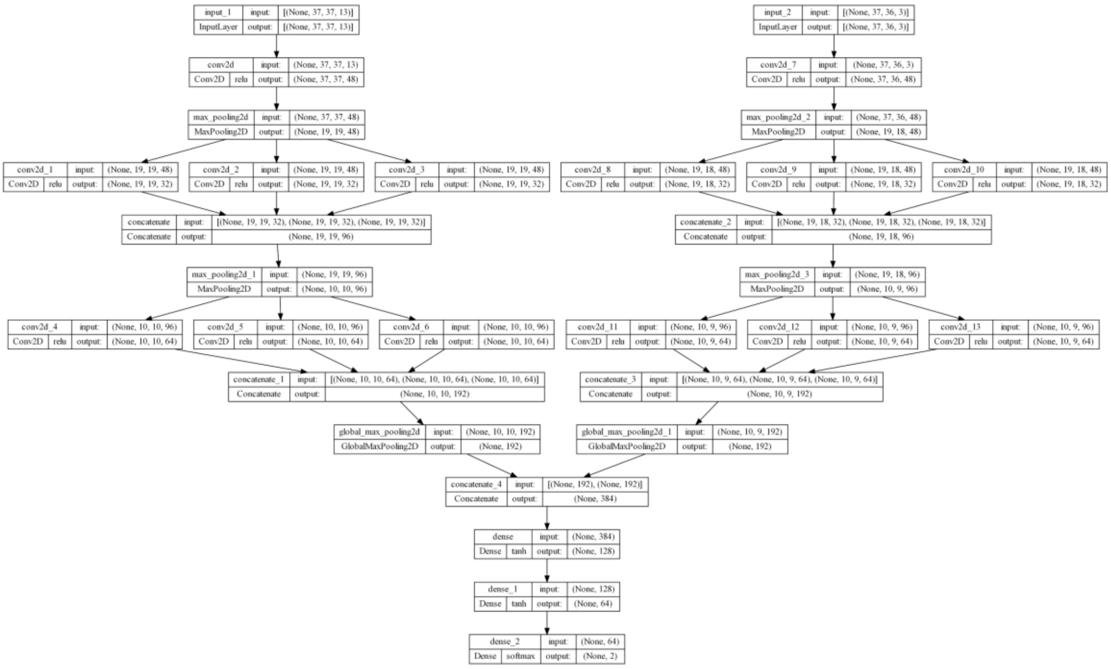

Fig. S5. SHAP values for the top 20 features of 2D descriptor.

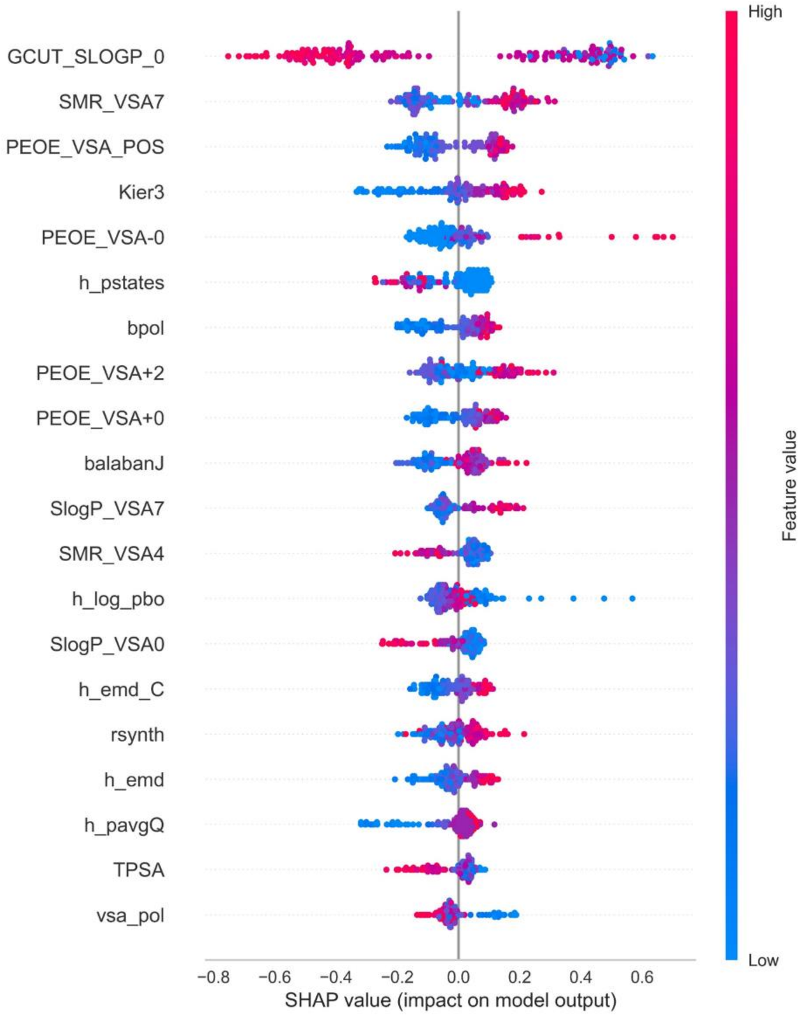

Fig. S6. SHAP values for the top 20 features of 2D+3D (MNDO) descriptor.

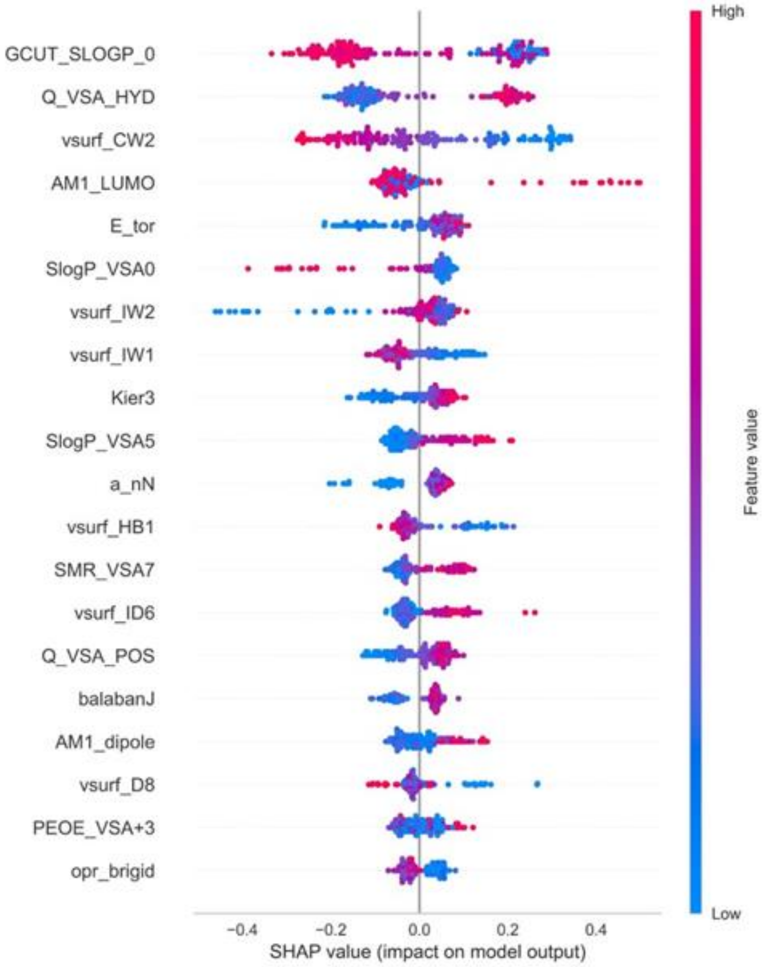

Fig. S7. SHAP values for the top 20 features of 2D+3D (PM3) descriptor.

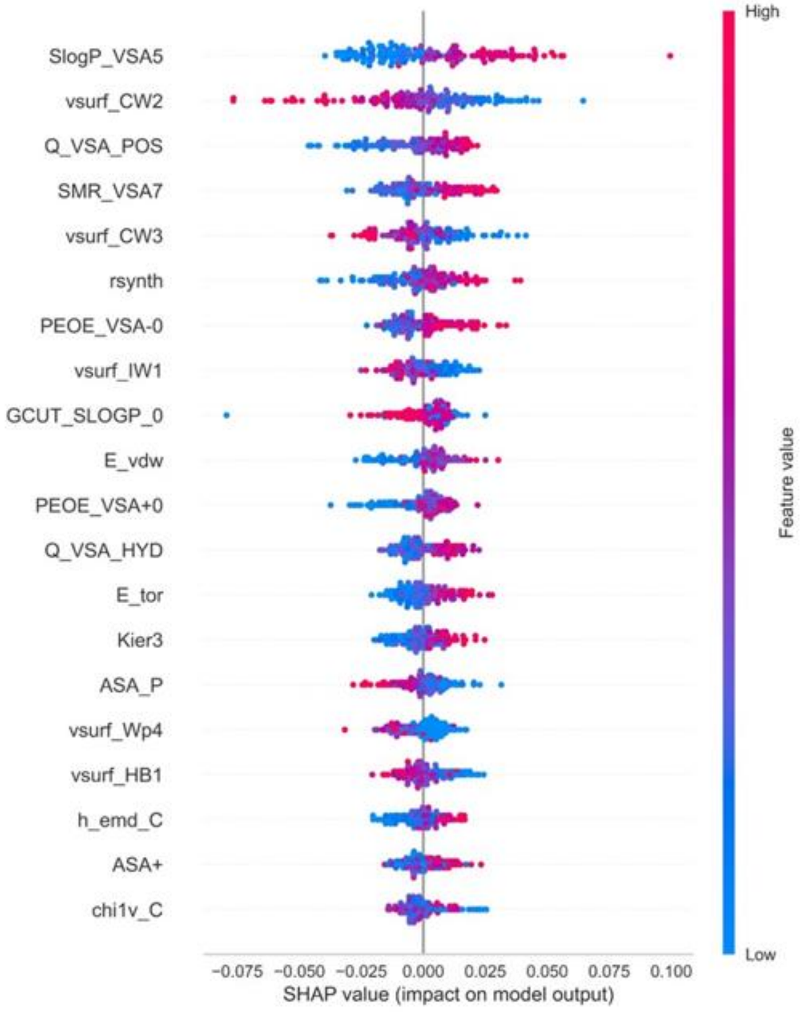

Fig. S8. MolMapNet top and bottom 50 important features.

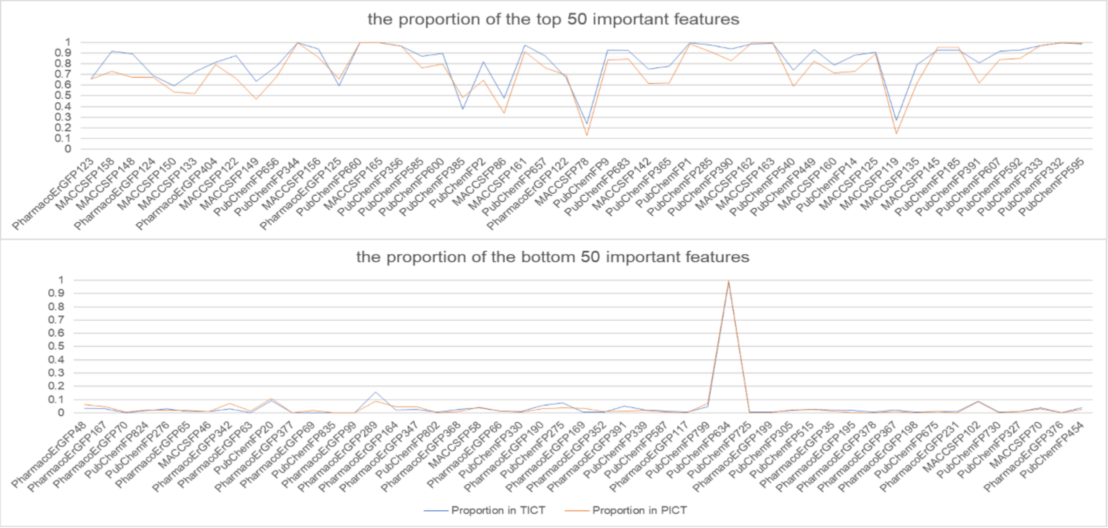

Fig. S9. Important MACCS fingerprint visualization.

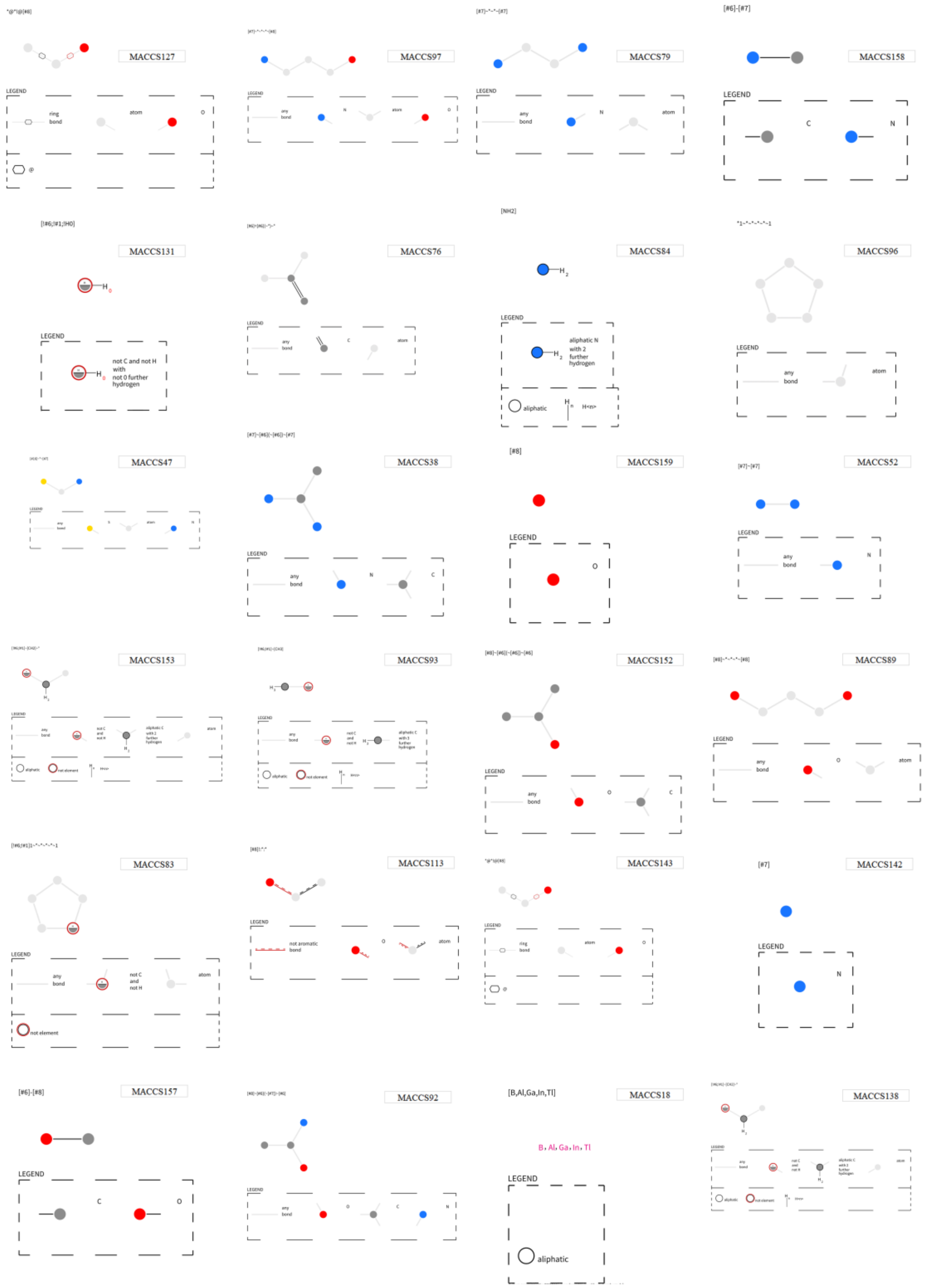

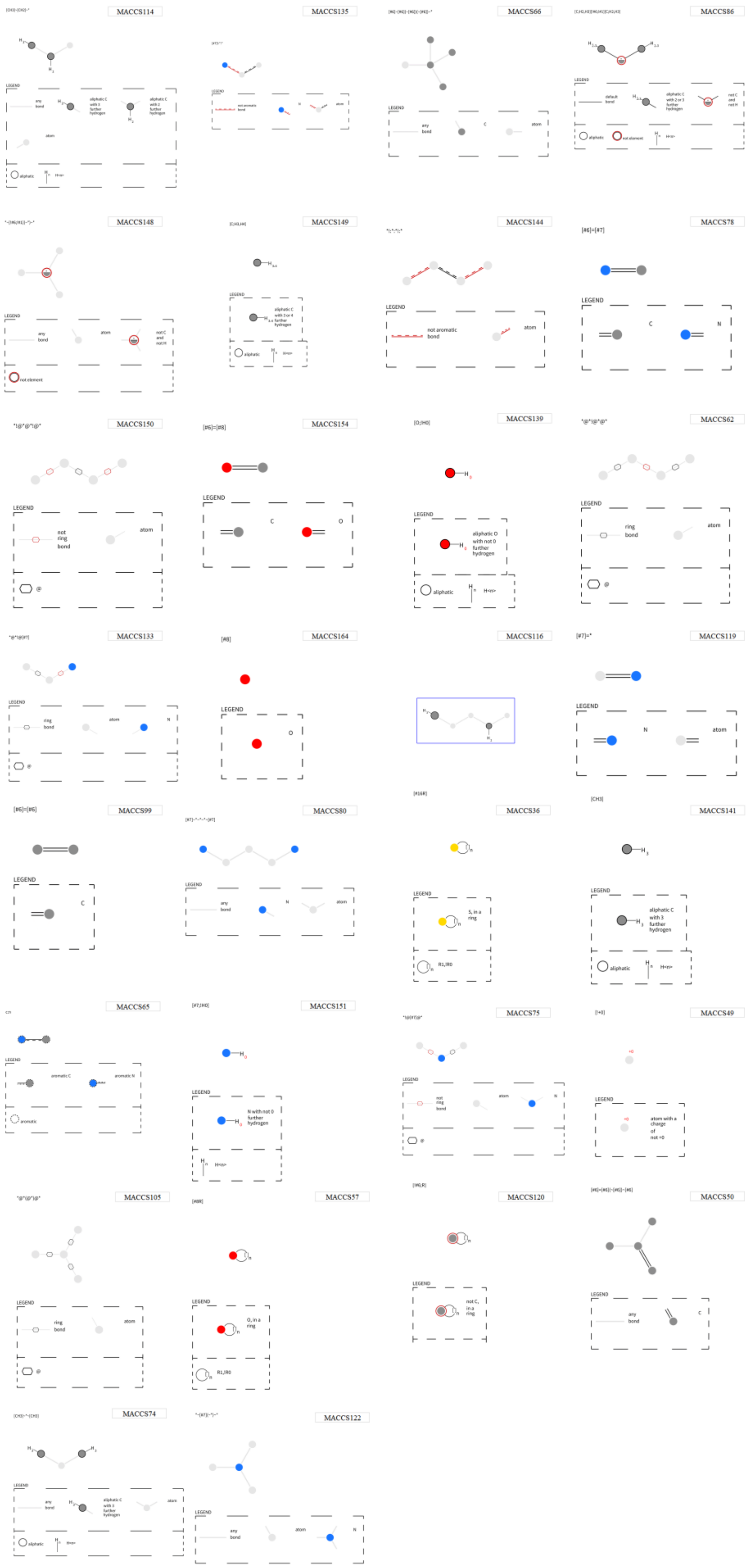
MACCS114
MACCS135
MACCS66
MACCS86
MACCS148
MACCS149
MACCS144
MACCS78
[#6]=[#7]
MACCS150
MACCS154
[#6]=[#8]
MACCS139
MACCS62
MACCS133
MACCS164
[#8]
MACCS116
MACCS119
MACCS99
[#6]=[#6]
MACCS80
MACCS36
MACCS141
MACCS65
MACCS151
MACCS75
MACCS49
MACCS105
MACCS57
MACCS120
MACCS50
MACCS74
MACCS122
LEGEND

Fig. S10. Predicted distribution of traditional chemical experience library after t-SNE dimensionality reduction.

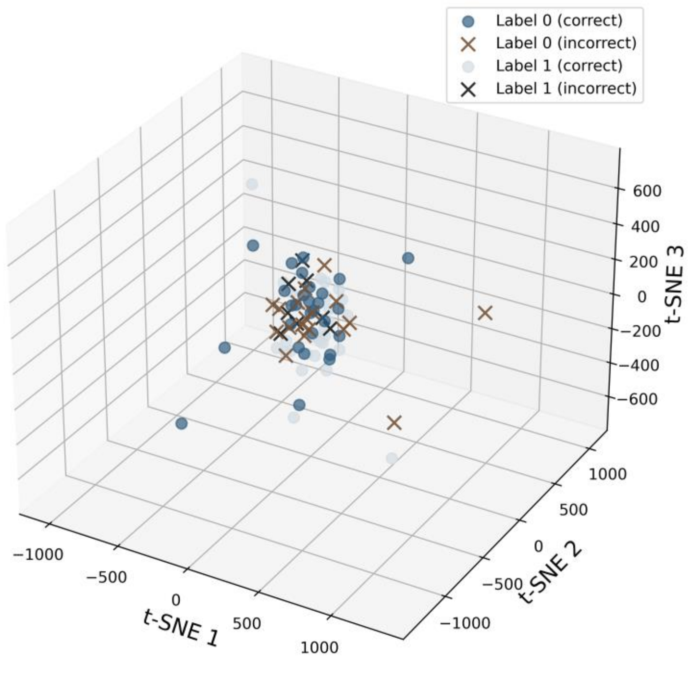

Fig. S11. Absorption spectra of Compound1 in different solvents.

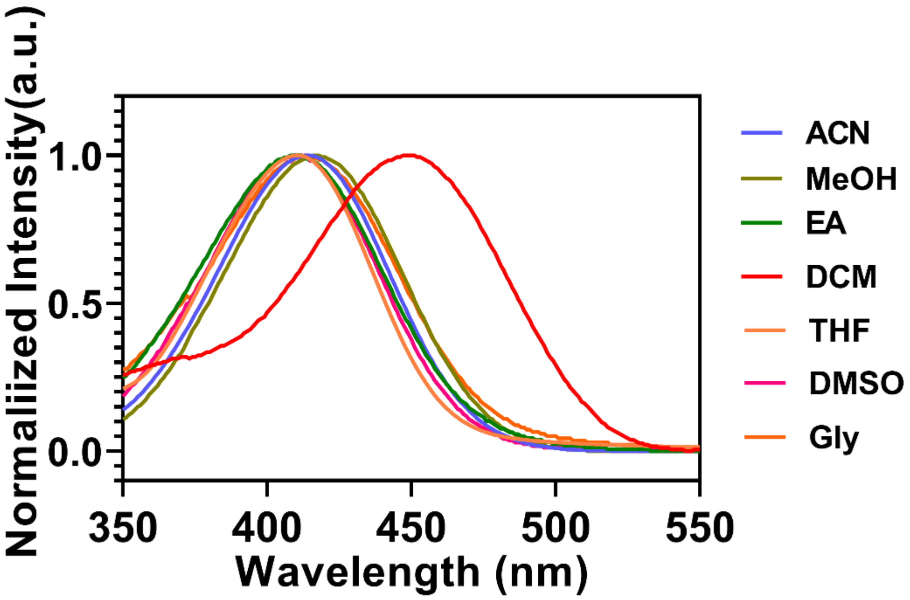

Fig. S12. Absorption spectra of Compound2 in different solvents.

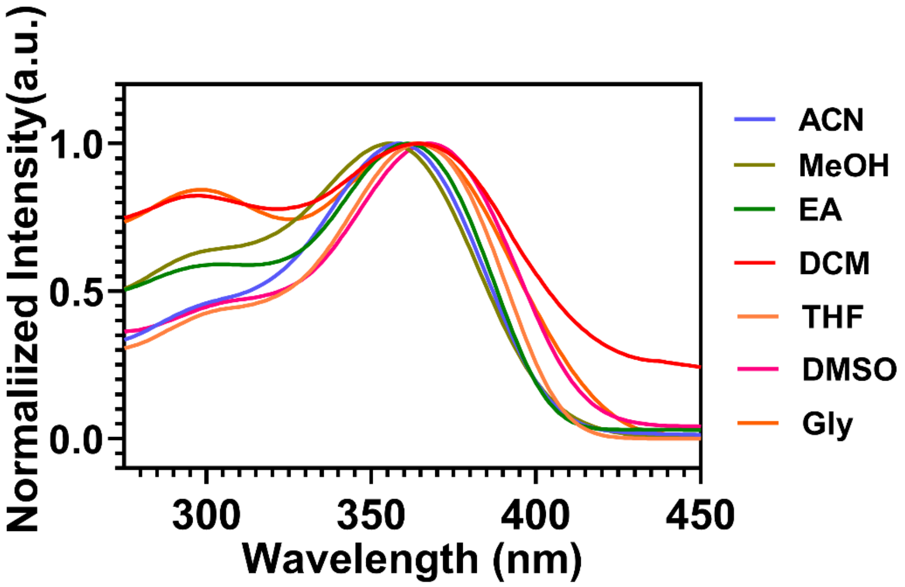

Fig. S13. Compound 1 in the HOMO and LUMO orbitals of the PICT state, and the HOMO and LUMO orbitals under complete charge separation in the TICT state.

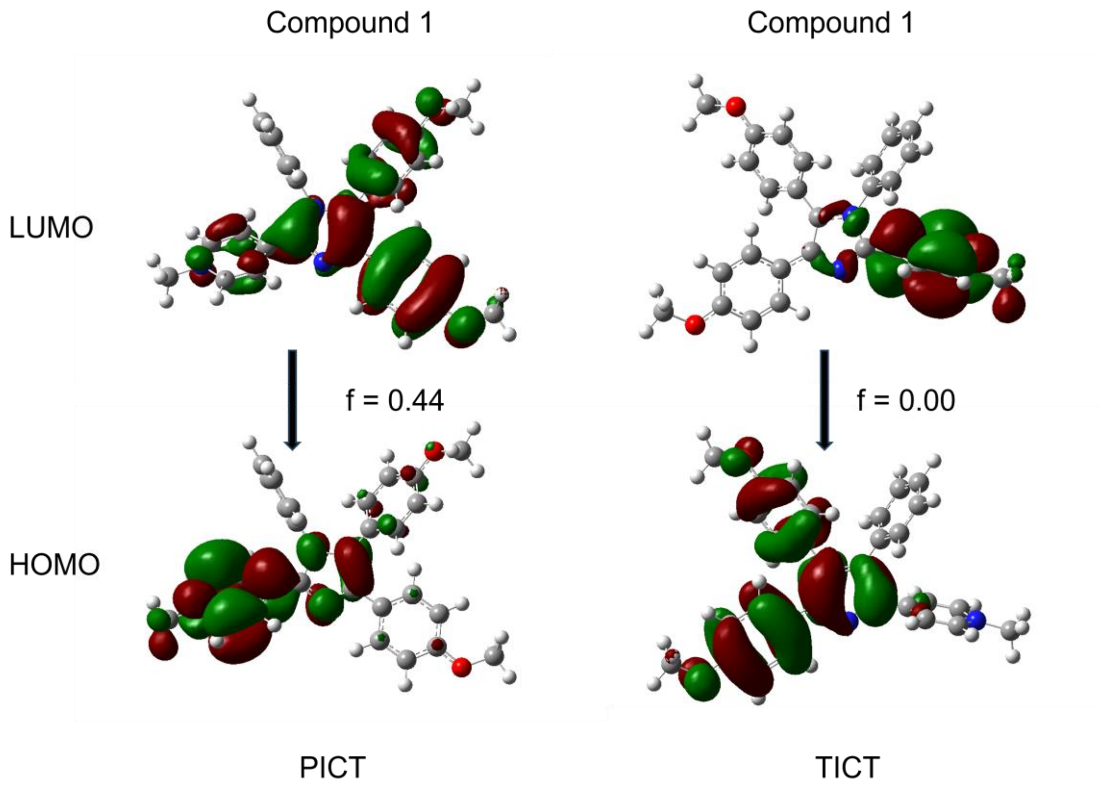

Fig. S14. Compound 2 in the HOMO and LUMO orbitals of the PICT state.

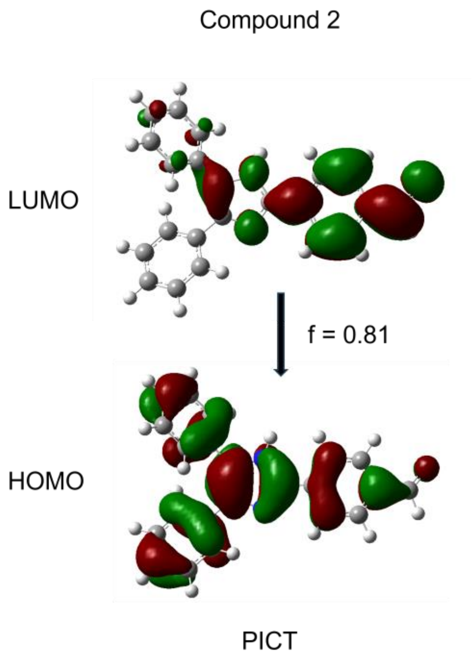

Fig. S15. Imaging of the viscosity response of Compound 1 and Compound 2 in cells. The scale was 20 μm.

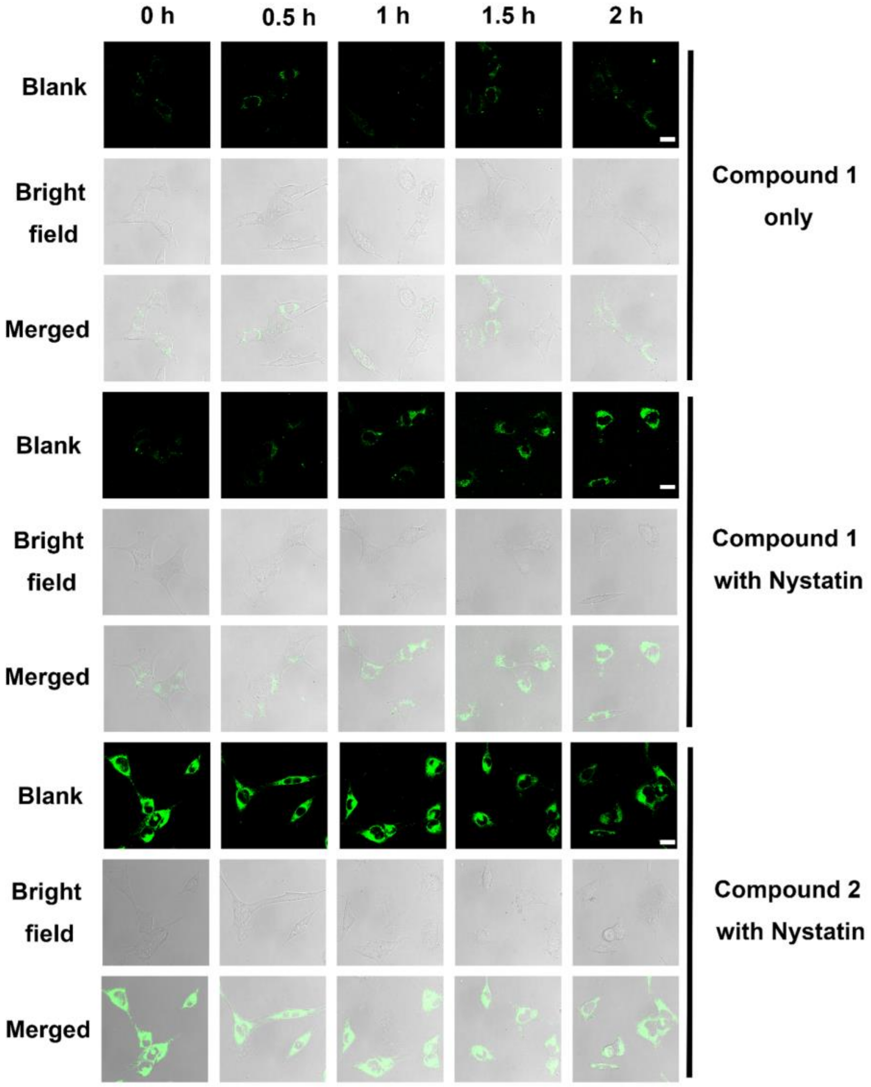

Fig. S16. ^{1}H-NMR spectrum of Compound 1 in $CDCl_3$.

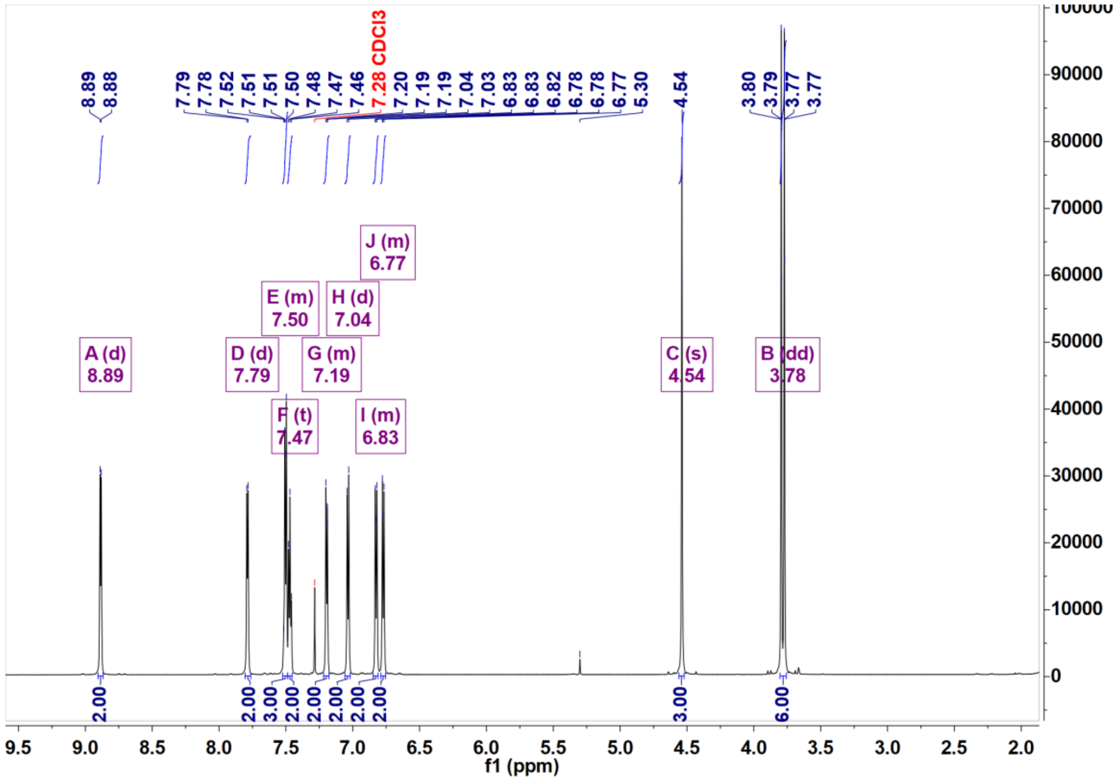

Fig. S17. ^{13}C-NMR spectrum of Compound 1 in $CDCl_3$.

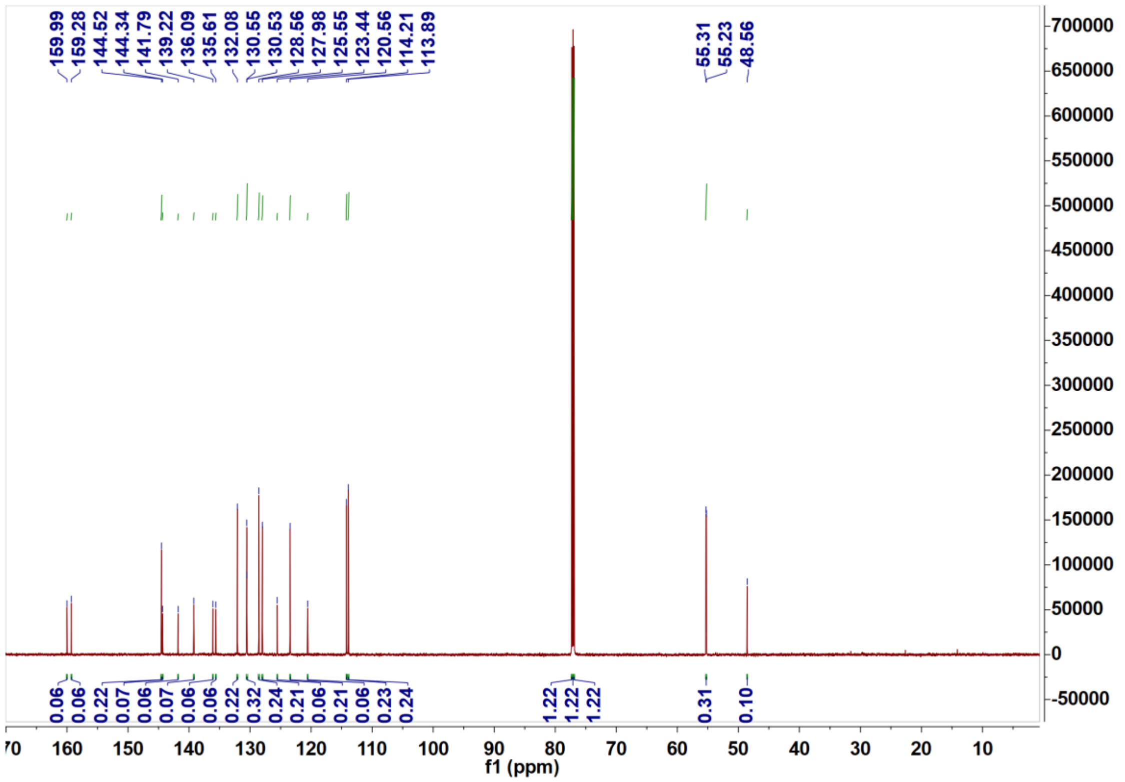

Fig. S18. HR-MS spectrum of Compound 1.

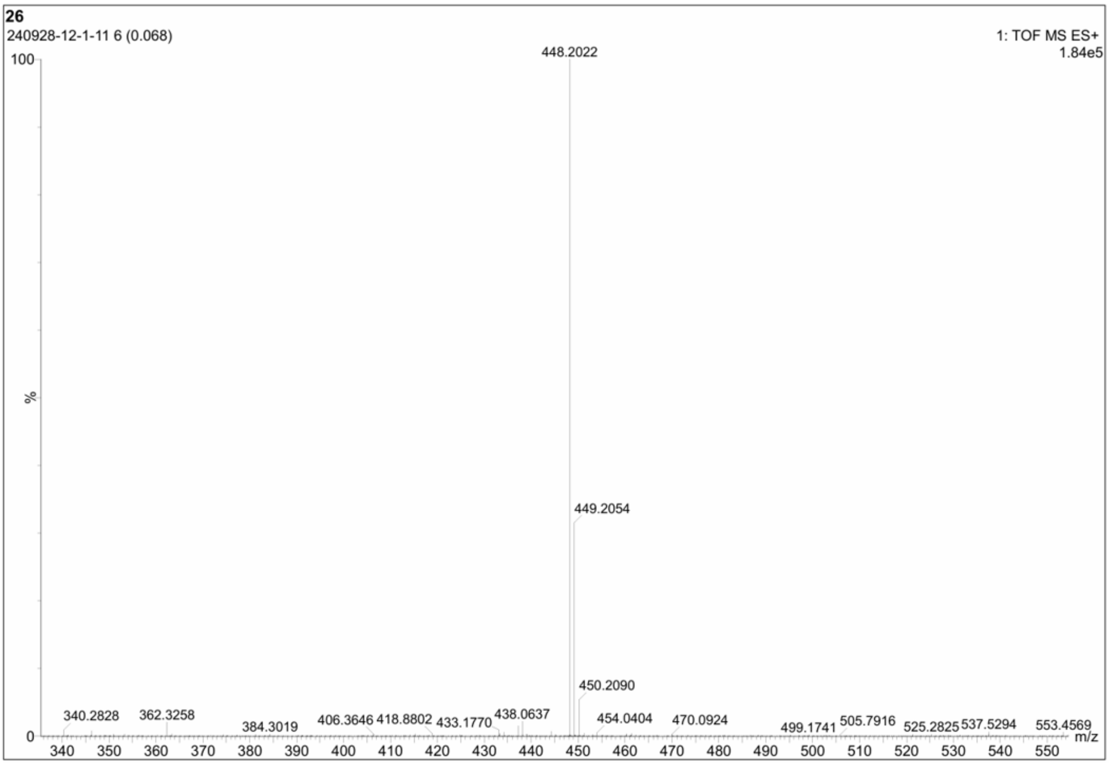

Supplement: Supplementary 1 — Supplementary materials for structure identification Figs. S1 to S18 Tables S1 to S14 [file research.1021.f1.pdf]
